# Alcohol consumption trajectories and associated factors in adult women: the Norwegian Women and Cancer study

**DOI:** 10.1093/alcalc/agaf005

**Published:** 2025-02-08

**Authors:** Fjorida Llaha, Idlir Licaj, Ekaterina Sharashova, Kristin Benjaminsen Borch, Marko Lukic

**Affiliations:** Department of Community Medicine, UiT The Arctic University of Norway, Tromsø, Norway; Department of Community Medicine, UiT The Arctic University of Norway, Tromsø, Norway; Department of Community Medicine, UiT The Arctic University of Norway, Tromsø, Norway; Department of Community Medicine, UiT The Arctic University of Norway, Tromsø, Norway; Department of Community Medicine, UiT The Arctic University of Norway, Tromsø, Norway

**Keywords:** alcohol, women, trajectories, cohort study, longitudinal analysis

## Abstract

**Aims:**

We described the age-specific trajectories of total alcohol consumption and the consumption of different types of beverages among adult Norwegian women as they age, and how these relate to education, lifestyle, and health-related factors.

**Methods:**

This study included 76 382 women aged 31–70 years who participated in at least two of the three Norwegian Women and Cancer (NOWAC) study surveys conducted in 1991–97, 1998–2003, and 2004–11. Group-based trajectory modeling was used to identify the trajectories of self-reported alcohol consumption. Multinomial regression models were used to fit the adjusted odds ratios (ORs) of the associations between education, lifestyle, health-related factors, and the trajectory membership. Analysis was stratified into two subcohorts: women aged 31–49 years and women aged 50–70 years at enrolment.

**Results:**

Five different trajectories of total alcohol consumption were identified among the two subcohorts: non-drinker stable (12.5%–23.6%), low stable (66.3%–60.1%), light increasing or light unstable (17.8%–12.1%), moderate to high or light to high (2.8%–2.7%), and high to moderate or moderate decreasing (.6%–1.4%). Trajectories were resembled by those of wine consumption. Compared to low stable drinkers, women who sustained or increased their total alcohol consumption showed higher ORs for higher education level, excellent self-rated health, former or current smoking status, and a body mass index (BMI) below 25 kg/m^2^.

**Conclusion:**

While most women in this study maintained stable low-light levels of alcohol consumption, certain groups—such as women with higher education and better health—were more likely to increase their drinking with age. Women can particularly increase their drinking around the retirement age. The increasing trends of total alcohol consumption were reflected by those of wine. These findings provide information into groups and beverages that could be targeted in alcohol-reducing interventions.

## Teaser

**Short Summary:** Most women consumed <10 g/day of alcohol and maintained their consumption stable. Approximately 2.7% showed increasing trends, particularly observed around the retirement age. Women who sustained or increased their alcohol consumption were more likely to be highly educated, to smoke, to report excellent health status and BMI below 25 kg/m^2^. Abstainers were more likely to report low education and poor health.

## Background

Alcohol consumption is an important global contributor to injuries, a number of non-communicable diseases and mortality (WHO 2024). Western countries, including Norway, are experiencing increases of alcohol consumption among women and older adults (Barry and Blow 2016, Keyes et al. 2019, McCaul et al. 2019, Bye and Moan 2020, Stelander et al. 2021). This is of concern because both women and older adults are more susceptible to the adverse effects of alcohol consumption (Barry and Blow 2014, Milic et al. 2018).

As alcohol consumption among women rises and the population ages in the western world, it is important to understand its trends among women and its underlying driving factors. Longitudinal studies with repeated measures are valuable in examining the development trajectories of alcohol consumption (Schulenberg and Maggs 2002). Trajectories of alcohol consumption can be informative in identifying critical life periods and at-risk groups earlier in life (Schulenberg and Maggs 2002), before the late adulthood when vulnerability to the negative effects of alcohol consumption increases (Barry and Blow 2014).

Longitudinal analyses of alcohol consumption show that most women drink <10 g/day and tend to maintain stable their consumption as they age (Han et al. 2019, Bassett et al. 2022, Mayén et al. 2022). However, considerable within-person changes occur in different segments of adulthood and are influenced by life events, such as pregnancy, transition to menopause or retirement (Milic et al. 2018, Agahi et al. 2022a, Muggli et al. 2022). Studies in adult populations have identified socioeconomic, health and lifestyle factors as predictors of longitudinal patterns of alcohol consumption. Overall, these studies have shown that adults with higher level of education, with better health status and who smoked have a greater likelihood of sustaining or increasing consumption as they age (Platt and Sloan 2010, Bobo et al. 2013, Halonen et al. 2017, Holton et al. 2019, Agahi et al. 2022a, Sidorchuk et al. 2022). Contrary, adults with poor health status have greater likelihood of abstaining or decreasing alcohol consumption as they age (Brennan et al. 2011, Holton et al. 2019, Sidorchuk et al. 2022).

Research gaps remain among the studies that investigated the longitudinal patterns of alcohol consumption in adults and their predictors (Moore et al. 2005, Platt and Sloan 2010, Brennan et al. 2011, Bobo et al. 2013, McEvoy et al. 2013, Halonen et al. 2017, Dobson et al. 2018, Holton et al. 2019, Skourlis et al. 2021, Agahi et al. 2022a, Agahi et al. 2022b, Sidorchuk et al. 2022, Baumann et al. 2022) (Supplementary Table S1). Most importantly, these studies have either included only men (Bobo et al. 2013), or did not conduct gender-specific analysis (Platt and Sloan 2010, Halonen et al. 2017, Dobson et al. 2018, Agahi et al. 2022a, Baumann et al. 2022, Sidorchuk et al. 2022), despite the known gender differences in alcohol drinking (Han et al. 2019, Bassett et al. 2022, Sidorchuk et al. 2022). Second, few studies have investigated separately the longitudinal consumption pattern of different beverages (Skourlis et al. 2021, Sidorchuk et al. 2022). Beverage-specific knowledge it can be an aid to develop specific public health policies and to predict drinking behaviors given their relationship with beverage choices (Dey et al. 2014, Loy et al. 2021). In Norway, overall there has been a shift in the composition of alcohol sales toward wine (Norwegian Institute of Public Health 2024). Various factors can influence beverage choices. For example, studies from Norway and Sweden have shown an increase in wine consumption as people age (Gustavsen and Rickertsen 2018, Kraus et al. 2022). Another study in Sweden observed beer as the preferred alcoholic beverage among individuals with lower socioeconomic position (Sidorchuk et al. 2022). Third, most studies have exanimated smoking, socioeconomic, and health factors as predictors of the longitudinal patterns of alcohol consumption (Moore et al. 2005, Brennan et al. 2010, Platt and Sloan 2010, McEvoy et al. 2013, Holton et al. 2019, Agahi et al. 2022a, Agahi et al. 2022b, Baumann et al. 2022). Hence, lifestyle factors such as body weight or physical activity have been less considered (Bobo et al. 2013, Halonen et al. 2017, Skourlis et al. 2021, Sidorchuk et al. 2022). Last, longitudinal studies from Sweden and Finland, which have historically shared similarities with Norway on alcohol restriction policies, are available (Halonen et al. 2017, Sidorchuk et al. 2022, Agahi et al. 2022a, Agahi et al. 2022b). However, some differences in alcohol consumption exist. For example, the increase in alcohol consumption among women has been particularly dominant in Norway (Tigerstedt et al. 2020). A common characteristic of the above studies is their focus in older adults, leaving a gap for the middle-aged population.

The aims of this explorative study, using data from the longitudinal Norwegian Women and Cancer (NOWAC) cohort which included women aged 31–70 years, were: (i) to identify the age-specific trajectories of total alcohol consumption in women; (ii) to investigate how these trajectories relate to education level, lifestyle, and health-related factors. Furthermore, the same analyses were extended to different type of alcoholic beverages.

## Methods

### Study design and participants

We used data from the NOWAC study, a nationwide prospective cohort that has been previously described in detail (Lund et al. 2008). All women were sampled randomly from the Norwegian National Population Register according to year of birth. From 1991 to 2007, a total of 172 478 women aged 30–70 years were enrolled into the study. In this cohort, approximately 90% of women were mothers. The response rate for those who were invited between 1991 and 1997 was 57%. A second and a third self-administrated questionnaire was sent to these women during 1998–2003 (response rate of 80%) and 2004–11 (response rate of 79%). Women who filled out at least two questionnaires and had complete data on alcohol consumption were included in this study (Supplementary Fig. S1). The interval period between the first and third completed questionnaire ranged from 8 to 15 years. In 2003–07 the cohort was expanded by inviting new participants, who were not yet invited by NOWAC to complete a third questionnaire. These women were not included in this study to ensure a more balanced data set in terms of the period of data collection, follow-up and number of completed questionnaires. This study was approved by the Regional Committees for Medical Health Research Ethics (REK) of North Norway (no. 488563) and has been performed in accordance with the standards of the Declaration of Helsinki. The NOWAC study was approved by Norwegian Data Inspectorate. All participants provided informed consent.

### Measures

The first completed questionnaire, administered during 1991–97, was noted as the enrolment measurement for the present study. Women self-reported the number of years of education, smoking status (never, former, current), physical activity level on a 10-increment scale from 1 to 10, weight (kg), height (cm), and if had or had ever had high blood pressure, diabetes, stroke, thrombosis in leg, or thigh, heart attack, heart failure (yes, no), and self-rated overall health (very poor, poor, good, excellent). In some questionnaires, and self-rated overall health (SRH very poor, poor, good, excellent). In some questionnaires, mental and physical SRH were asked instead of the overall SRH. Women who reported equal categories for physical and mental SRH were assigned to the corresponding category of overall SRH. We merged “poor” and “very poor” into one “poor” category as very few women reported “very poor” SRH. Given the small number of women with history of comorbidities, having at least one of the above comorbidities (yes, no) was considered. Body mass index (BMI) was calculated (kg/m^2^). BMI was categorized into <25 and ≥25 kg/m^2^, years of education completed at the time of study enrolment were categorized into ≤9, 10–12, and ≥13, physical activity level into low (score 1–5), and moderate-high (6–10 score). In each questionnaire women reported their age at first and last birth. If a woman’s age matched her age at her first or last birth, we assumed that the alcohol recall period (average alcohol intake over the last 12 months) coincided with pregnancy or breastfeeding periods. To account for this, a new variable labeled “alcohol recall period matched with pregnancy/breastfeeding periods (yes, no)” was created and was used for sensitivity analysis. Regarding alcohol consumption, women were first asked if they were alcohol abstainers. If not, they were asked to report the frequency of the units of wine, beer, spirits, and liqueurs consumed over the past 12 months. For each alcoholic drink, women could choose between the following answers: never/seldom, 1 unit /month, 2–3 units/month, 1 unit/week, 2–4/week, 5–6 units/week, 1 unit/day, ≥2 units/day. A midpoint value for each category was assigned to calculate the number of units consumed per day. Grams of alcohol consumed per day from alcoholic drinks were calculated based on estimated average unit volume and alcohol content for each type of beverage, using values from the Norwegian Weights and Measurement Table (Hjartåker et al. 2007). A zero value was assigned to the never/seldom answers. A total gram of alcohol consumed per day was the primary dependent variable in this study. The secondary dependent variables were alcohol consumption from wine, beer, and spirits/liqueurs.

### Statistical analysis

Given the exploratory nature of the study and limited knowledge on alcohol consumption trajectories in women, we used a data-driven approach to identify the longitudinal patterns. To align with the World Health Organization (WHO 2023) and the Norwegian guidelines (Norwegian Directorate of Health 2024), which state there is no scientifically valid “safe” level of alcohol consumption and recommend drinking as little as possible, we avoided modeling trajectories based on predefined risk categories. We used the semi-parametric group-based trajectory modeling (GBTM) to explore groups of women with similar trajectories of alcohol consumption. The GBTM fits longitudinal data to a number of discrete latent trajectories and accounts for missing data via maximum likelihood (Nagin and Odgers 2010, Nagin et al. 2018). Alcohol quantity data (g/day) were used to model the trajectories of alcohol consumption as a function of age. The median age of study sample at each wave of data collection was used as indicator of time in the GBTM. Trajectories were modeled separately in two subcohorts defined by age at enrolment, 31–49 years, and 50–70 years. The age stratification was done to consider the influence of life events in women during different segment of adulthood. The final grouping 31–49 and 50–70 years was determined after observing very similar trajectories between the age groups 31–39 and 40–49 years. To ensure an adequate sample size, the age groups 50–59 years and 60–70 years, representing respectively 15% (*n* = 11 456) and 8% (*n* = 6362) of the study sample, were merged. In the younger subcohort (31–49 years) 75% of women returned the first questionnaire during 1991–92 and 15% between 1996 and 1997. The second questionnaire was returned during 1998–2003, and the third during 2004–11. In the older subcohort (50–70 years), all women returned the first questionnaire during 1996–97. The second questionnaire was returned during 2002–03, and the third during 2010–11. We excluded sixteen women (Supplementary Table S2) in the younger subcohort with total alcohol consumption >100 g/day in order to improve the convergence and stability of the GBTM model. Following Nagin and colleagues (Nagin and Odgers 2010, Nagin et al. 2018), the optimal number and polynomial of trajectory classes was established considering the Bayesian information criterion (BIC) statistics, average posterior probabilities of assignment (>.70), proportion of a sample assigned to a certain trajectory class close to the proportion estimated from the model, and a reasonably narrow confidence interval (CI) around each trajectory (Supplementary Methods 1). Women were assigned to the trajectory groups for which their posterior probability predicted from the final model was highest. Individual alcohol consumption trajectories by each group were plotted to visually judge that the GBTM classified individuals optimally (Supplementary Figs S2–S9). The groups of alcohol trajectories were labeled according to their most prominent trajectory of consumption. The GBTM model was fitted with traj package in Stata® 17.0. The Stata codes for the final GBTM models are available in Supplementary Methods 1.

 Associations between education level, smoking status, SRH, BMI, physical activity level, history of comorbidities at enrolment and alcohol consumption trajectories membership were estimated as odds ratios (ORs) with 95% CIs using multinomial logistic regression. We fitted separate models for each independent variable. We adjusted the regression models for age, education level, smoking status, and SRH, as important predictors of alcohol consumption (Platt and Sloan 2010, Bobo et al. 2013, Halonen et al. 2017, Holton et al. 2019, Agahi et al. 2022a, Sidorchuk et al. 2022) and which are commonly adjusted for in previous longitudinal studies (Bobo et al. 2013, McEvoy et al. 2013, Dobson et al. 2018, Holton et al. 2019, Skourlis et al. 2021, Baumann et al. 2022). This adjustment enabled us to estimate the association of each characteristic independently of case-mix variations and to compare the identified trajectories in this study with those from other studies, thereby enhancing the validity of our trajectories computed through a data-driven approach. We did not adjust for parity or enrolment year in the study because these variables did not improve the model fit and did not change the results. The regression models with each type of beverage as dependent variables were further adjusted for the trajectory memberships of other beverages. In all models, the “low stable” trajectory was the omitted category of the dependent variable. Our rationale for not omitting the non-drinker trajectory was that the findings from these analyses could help to understand the elevated health risks, for example the higher risk for coronary heart disease and mortality, found among people who abstain alcohol (Licaj et al. 2016, O’Neill et al. 2018). Missing data for history of comorbidities were considered as “no” after testing that missing and non-missing data gave similar results from the regression analysis. The regression analysis was done by excluding the missing data of the independent variable and covariates, ranging from 1.5% to 14.9% (Supplementary Table S2).

As sensitivity analyses, we remodeled the trajectories of total alcohol consumption after excluding women who were potentially pregnant or breastfeeding during alcohol data collection or excluding women who died before completing the third questionnaire. The date of death was retrieved from the Norwegian Cause of Death Registry. Further, we remodeled the trajectories of total alcohol consumption and spirits after removing the consumption data for liqueurs, since liqueurs data were not collected in all the questionnaires.

## Results

Descriptive characteristics of the 76 382 women stratified by subcohorts are presented in Table 1. Mean age at enrolment in the younger subcohort (31–49 years at enrolment) was 41.5 years and 57.6 years in the older subcohort (50–70 years at enrolment). Compared to older women, younger women reported higher level of education and physical activity, lower BMI, better health status, and slightly higher alcohol consumption at enrolment.

**Table 1:** Characteristics at enrolment of the study sample stratified by subcohorts defined by the age at enrolment. The NOWAC Study 1991–2011.

		**All**	**31–49 years**	**50–70 years**
N (%) of women		76 382	58 564 (76.7)	17 818 (23.3)
Age, mean (SD), years		45.3 (8.4)	41.5 (4.6)	57.6 (5.7)
Total alcohol in g/day, median (IQR)		1.6 (0, 3.1)	1.7 (0, 4.2)	1 (0, 3.1)
Alcohol from wine in g/day, median (IQR)		0.8 (0, 1.6)	0.8 (0, 1.6)	0.4 (0, 1)
Alcohol from beer in g/day, median (IQR)		0 (0, 1.2)	0 (0, 1.2)	0 (0, .6)
Alcohol from spirits/liqueurs in g/day, median (IQR)		0 (0, .9)	0 (0, .9)	0 (0, .5)
Years of education, *n* (%)				
≤9		18 749 (24.6)	11 748 (20.1)	7001 (39.3)
10–12		25 405 (33.3)	20 602 (35.2)	4803 (26.9)
≥13		28 746 (37.6)	24 632 (42.1)	4114 (23.1)
Missing		3482 (4.6)	1582 (2.7)	1900 (10.7)
Smoking status, *n* (%)				
Never		27 308 (35.8)	19 801 (33.8)	7504 (42.1)
Former		22 926 (30)	17 359 (29.6)	5567 (31.2)
Current		25 079 (32.8)	20 528 (35.1)	4551 (25.5)
Missing		1069 (1.4)	876 (1.5)	193 (1.1)
Self-rated health, *n* (%)				
Poor		4230 (5.5)	2953 (5)	1277 (7.2)
Good		39 277 (51.4)	29 214 (49.9)	10 063 (56.5)
Excellent		21 990 (28.8)	18 290 (31.2)	3700 (2.8)
Missing		10 885 (14.3)	8107 (13.8)	2778 (15.6)
BMI, mean (SD), kg/m^2^		23.6 (3.6)	23.1 (3.4)	25.1 (3.8)
BMI categories, *n* (%)				
<25 kg/m^2^		54 152 (70.9)	44 446 (75.9)	9706 (54.5)
≥25 kg/m^2^		20 873 (27.3)	13 142 (22.4)	7731 (43.4)
Missing		1357 (1.8)	976 (1.7)	381 (2.1)
Physical activity level, *n* (%)				
Low		34 817 (45.6)	26 178 (44.7)	8639 (48.5)
Moderate-High		34 579 (45.3)	27 698 (47.3)	6881 (38.6)
Missing		6986 (9.2)	4688 (8)	2298 (12.9)
History of comorbidities, *n* (%)				
No		67 944 (88.9)	54 156 (92.5)	13 788 (77.4)
Yes		8438 (11.1)	4408 (5.5)	4030 (22.6)

SD, standard deviation. IQR, interquartile range. BMI, body mass index.

### Trajectories of alcohol consumption

**Figure 1:**
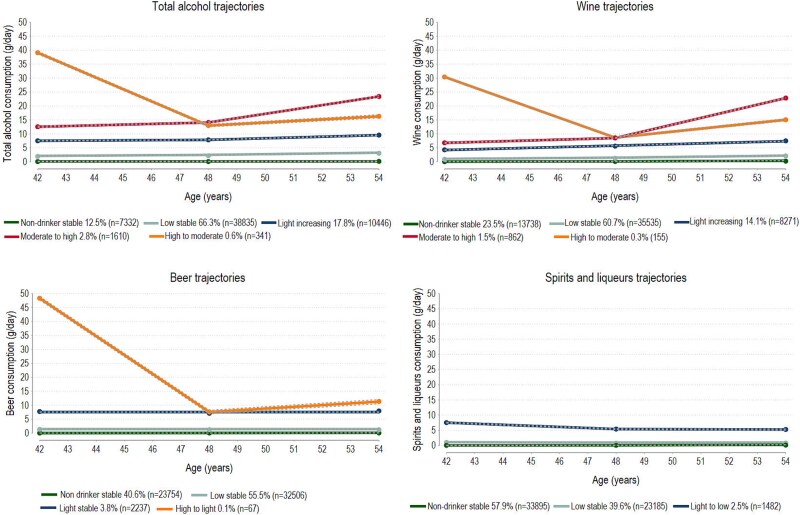
Mean predicted trajectories with 95% CIs (dash lines) of alcohol consumption (g/day) from total alcohol and alcohol-specific beverages in women aged 31–49 years at enrolment.

**Figure 2:**
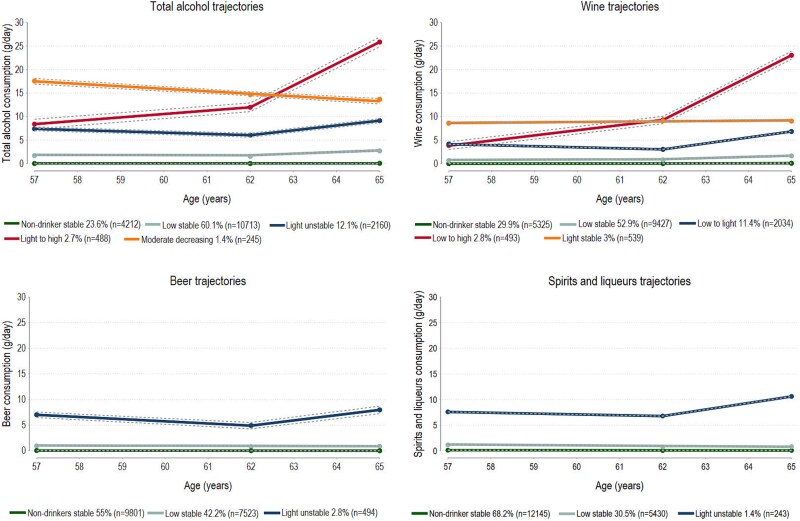
Mean predicted trajectories with 95% CIs (dash lines) of alcohol consumption (g/day) from total alcohol and alcohol-specific beverages in women aged 50–70 years at enrolment.

The optimal GBTM model identified five distinct trajectories for total alcohol consumption, varying slightly between the two subcohorts (Figs 1 and 2). In both subcohorts, non-drinker stable, low stable (<5 g/day of alcohol) and light drinking (5–10 g/day of alcohol) trajectories were identified. The low-stable trajectory was the most common trajectory (60.1%–66.3%). In both subcohorts, a decreasing trajectory was identified. In the younger subcohort, it was labeled as high to moderate, marked by a decrease in alcohol consumption from 39 to 16 g/day at the end of follow-up. In the older subcohort, it was labeled as moderate decreasing, with alcohol consumption decreasing from 18 to 14 g/day. An increasing trajectory was also found in both subcohorts. In the younger subcohort, it was labeled as moderate to high, marked by an increase of alcohol from 13 to 24 g/day at the end of follow-up. In the older subcohort, it was labeled as light to high, with alcohol consumption increasing from 8 to 26 g/day by the end of follow-up. The distribution of baseline characteristics according to the trajectory groups are given in Supplementary Table 3 and 4.

Trajectories of wine consumption closely mirrored those of total alcohol. For beer, and spirits/liqueurs most women belonged to the non-drinker stable and low stable trajectories, and few (1.4%–2.5%) belonged to the light drinking trajectories. A fourth trajectory group for beer was identified only in the younger subcohort. This was labeled high to light with alcohol consumption from beer decreasing from 48 to 12 g/day.

### Education level and the trajectories of alcohol consumption

**Figure 3:**
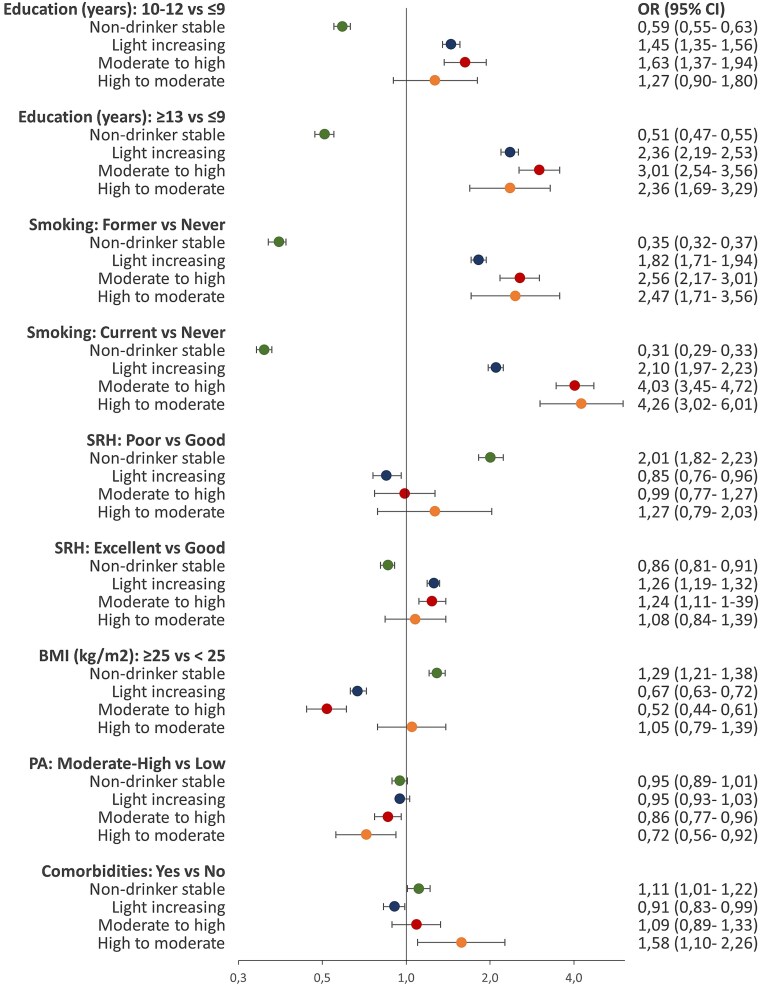
The ORs with 95% CI of the adjusted associations between characteristics at enrolment and total alcohol consumption trajectories in women aged 31–49 years at enrolment. Figure footnotes: The reference category for the dependent variable was low stable trajectory. SRH, self-rated health. PA, physical activity level

**Figure 4:**
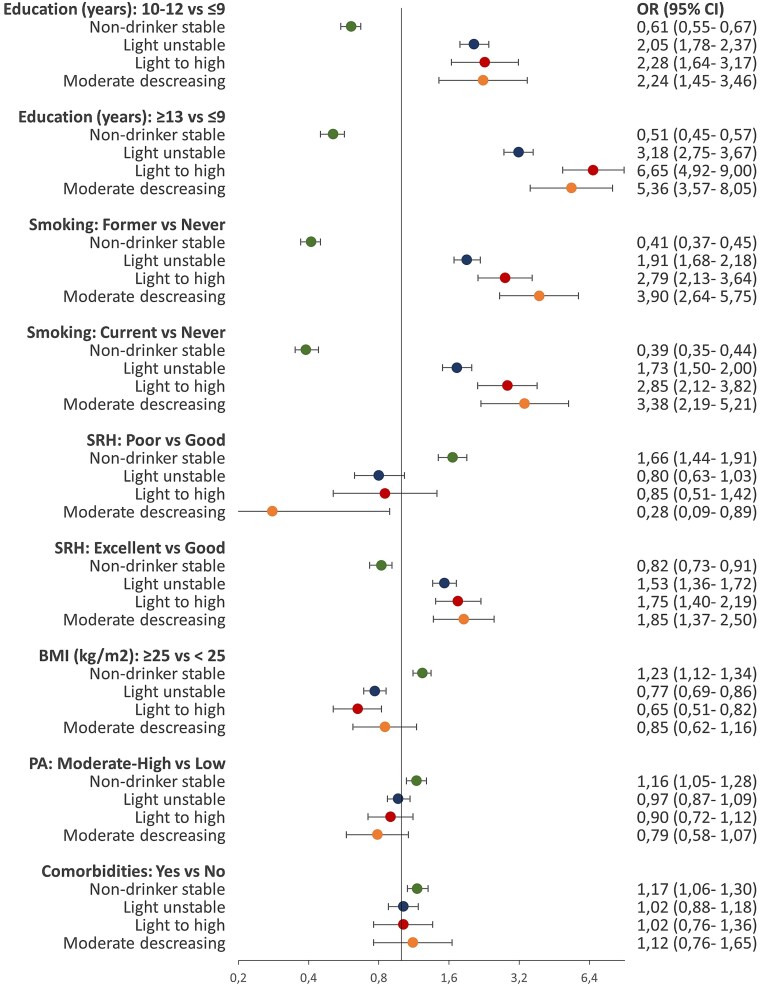
The ORs with 95% CI of the adjusted associations between characteristics enrolment and total alcohol consumption trajectories in women aged 50–70 years at enrolment. Figure footnotes: The reference category for the dependent variable was low stable trajectory. SRH, self-rated health. PA, physical activity level

Compared to women with low level of education, women with higher levels of education showed higher likelihood of belonging to the light, moderate, and high drinking trajectories of total alcohol and wine in both subcohorts. The ORs for total alcohol ranged from 1.45 to 6.65 (Figs 3 and 4) and for wine ranged from 1.87 to 8.40 (Supplementary Figs S10 and S13). The same pattern of association was observed for light drinking trajectories of beer in both subcohorts (Supplementary Figs S11 and S14) and the light drinking trajectories of spirits/liqueurs in the older subcohort (Supplementary Fig. S15) In contrast, younger women with higher levels of education showed lower likelihood of belonging to the light drinking trajectory of spirits/liqueurs and high to light drinking trajectory of beer (Supplementary Figs S11 and S12).

### Health-related factors and the trajectories of alcohol consumption

Compared to women with good SRH, women with poor SRH showed higher likelihood of belonging to the non-drinking trajectories (Figs 3 and 4). This trend was consistent across subcohorts and all beverages (ORs range 1.37–2.01) (Figs 3 and 4, Supplementary Figs S10–S15). We found the same pattern of association for the beer trajectory marked by decreases of alcohol consumption from high to light levels (Supplementary Fig. S11). Excellent SRH was associated with higher likelihood of belonging to the total alcohol and wine drinking trajectories in both subcohorts. The ORs for these associations ranged from 1.08 to 1.85 for total alcohol trajectories (Figs 3 and 4) and from 1.32 to 2.00 for wine trajectories (Supplementary Figs S10 and S13). The same trend was found for light drinking trajectories of beer and spirits/liqueurs in the older subcohort but not in the younger subcohort (Supplementary Figs S11, S12, S14, and S15). Women who reported history of comorbidities compared to women without history of comorbidities showed higher likelihood of belonging to the non-drinking trajectories. The ORs of these associations ranged from 1.01 to 1.21 (Figs 3 and 4, Supplementary Figs S10–S15). Younger women with history of comorbidities showed also higher likelihood of belonging to the trajectories characterized by steep decreases of total alcohol and beer consumption. These trajectories involved transitioning from high to moderate consumption of total alcohol (OR = 1.58, 95% CI = 1.10–2.26, Fig. 3) and from high to light consumption of beer (OR = 2.36, 95% CI = 1.15–4.81, Supplementary Fig. S11).

### Lifestyle factors and the trajectories of alcohol consumption

Compared to never smokers, former and current smokers showed lower likelihood of belonging to the non-drinking trajectories and higher likelihood of belonging to the light, moderate, and high drinking trajectories in both subcohorts (ORs range 1.73–4.26, Figs 3 and 4), and across different beverages (Supplementary Figs S10–S15). In the younger subcohort, for the same level of alcohol consumption (light level), smoking showed stronger association for spirits/liqueurs (OR = 2.73) than wine (OR = 1.71) or beer consumption (OR = 1.66) (Supplementary Figs S10–S12). Women in both subcohorts with BMI ≥25 kg/m^2^ compared to women with BMI <25 kg/m^2^ showed higher likelihood of belonging to the non-drinking trajectories of total alcohol, wine, and beer (ORs range 1.12–1.37). They showed also higher likelihood in belonging to the high to light beer trajectory in the younger subcohort and to light unstable spirits/liqueurs trajectory in the older subcohort (Supplementary Figs S11 and S15). Compared to women with low physical activity level, those with moderate-high physical activity level were less likely to belong to the trajectories with the highest level of alcohol consumption, including moderate to high (OR = .86, 95% CI: .77, .96), high to moderate (OR = .72, 95% CI: .56, .92), and moderate decreasing (OR = .79, 95% CI: .58, 1.07). The same pattern of association was found for the drinking trajectories of beer, and spirits/liqueurs with ORs ranging from .50 to .81 (Supplementary Figs S11, S12, S14, and S15). Regarding wine, this pattern was found only for moderate to high wine trajectory in the younger subcohort (OR = .85, 95% CI: .73, .99, Supplementary Fig. S10).

### Sensitivity analyses

Similar trajectories of total alcohol consumption were identified when excluding women who died before completing the third questionnaire (*n* = 566 in the younger subcohort and *n* = 929 in the older subcohort) or excluding women who were pregnant or breastfeeding during alcohol data collection (*n* = 470 in the younger subcohort) (Supplementary Figs S16 and S17). Results of regression analyses using these trajectories were not attenuated (data not shown). The trajectories of total alcohol consumption and spirits remained similar after removing the consumption data for liqueurs (Supplementary Fig. S18). In this cohort, the consumption of liqueurs was notably low. Among women who were surveyed about liqueur consumption and reported drinking liqueurs, the 95th percentile of consumption was 1.13 g/day.

## Discussion

### Overall findings

In this population-based longitudinal cohort study of 8–15 years of follow-up, we investigated the trajectories of alcohol consumption in Norwegian women aged 31–49 years and 50–70 years old. Most women (around 65%) followed a low-stable alcohol consumption trajectory (drinking <5 g/day), while approximately 2.7% displayed increases in alcohol consumption. Overall, trajectories of wine consumption resembled those of total alcohol. Women who sustained or increased their alcohol consumption showed higher ORs for higher education level, excellent SRH, former or current smoking status, and lower ORs for a BMI ≥ 25 kg/m^2^.

### Comparison with other data / studies

Our trajectory models, based on self-reported alcohol consumption data collected from 1991 to 2011, reflect the trends shown in Norwegian alcohol sales from 1991 to 2010, with sales of beer and spirits that somewhat declined and those of wine arising (Norwegian Institute of Public Health 2024). Alcohol sales increased from 1994 to 2008 and declined from 2008 to 2014. Overall, a period effect cannot be excluded as a contributor to the increased trends observed by the end of the study. This is particularly relevant for younger women, as 90% of them provided their last alcohol measurements between 2004 and 2007. Older women provided their last alcohol measurement between 2010 and 2011. The increased trend was more pronounced among older women, suggesting a particular age effect in this group. The pronounced rise of alcohol consumption observed around the retirement age (62–65 years), may be attributed to alcohol use in response to physical and mental distress (Platt and Sloan 2010, Britton et al. 2015), or retirement transition in itself (Syse et al. 2017). Our results align with prior population-based studies, showing that most women during adulthood follow low or light stable trajectories and that increased or decreased trends are more likely to occur at moderate or high alcohol consumption levels (>10 g/day) (Han et al. 2019, Bassett et al. 2022). Wine was the primary contributor to total alcohol consumption in our study, supporting the evidence from a Swedish study that a high proportion of wine consumption is related to light-moderate drinking patterns (Kraus et al. 2022).

The relationship between social gradients and alcohol consumption is complex. While higher educated adults are more likely to drink alcohol, they are less likely to drink heavily (Halonen et al. 2017, Holton et al. 2019). In this study, higher educated women followed stable or increases trends of total alcohol and wine consumption, alighting with several longitudinal studies (Platt and Sloan 2010, Holton et al. 2019, Agahi et al. 2022a, Baumann et al. 2022). Higher educated women were also more likely to follow decreases trends in alcohol consumption, possibly due to their greater health risk awareness related to high levels of alcohol consumption (Doyle et al. 2023). Among younger women in our study, those with higher education levels were more likely to follow the wine trajectory with the highest level of alcohol, while the opposite was seen for the beer trajectory with the highest level of alcohol. This is consistent with the Swedish study which found that individuals with lower socioeconomic status were more likely to drink beer at heavy levels, a pattern not observed for the heavy drinking levels of wine (Sidorchuk et al. 2022). One difference between younger and older women was the relationship of higher education level with drinking trajectories of spirits/liqueurs observed only among older women.

Our findings that women with excellent SRH were more likely to drink alcohol are consistent with several longitudinal studies (Platt and Sloan 2010, Bobo et al. 2013, Holton et al. 2019, Sidorchuk et al. 2022). We add to the literature that while younger women with excellent SRH were more likely to belong to the drinking trajectories of total alcohol and wine, this pattern of association did not extend to the drinking trajectories of beer and spirits/liqueurs. We also found that younger women who reported common comorbidities were more likely to belong to the trajectories with decreasing trends of total alcohol, wine, and beer consumption.

In our study, women who smoked were more likely to belong to the light, moderate, high drinking trajectories. Across the light drinking trajectories, we noted among younger women that this pattern of association was stronger for spirits/liqueurs compared to wine and beer consumption. A similar pattern of association was reported by a Greek study (Skourlis et al. 2021). Women who were current smokers tended to follow increasing and decreasing alcohol consumption trends. A study from US also found that the declined drinking rates as people age were faster among smokers compared to non-smokers (Moore et al. 2005). Women who belonged to the decreasing trajectory (decreasing from high to moderate levels) were also more likely to report comorbidities. This might be a reflection of health problems from both smoking and high alcohol consumption. Two previous longitudinal studies showed no influence of BMI on alcohol consumption over time among women and men (Halonen et al. 2017) and women only (Soler-Vila et al. 2019). Another longitudinal study showed decreases in total alcohol, wine, and beer consumption over time among overweight and obese adults, with no changes in spirits consumption (Skourlis et al. 2021). In our study, women with a BMI ≥25 kg/m^2^ were less likely to belong to the drinking trajectories of light stable or increasing consumption of total alcohol, wine, and beer. With regard to spirits/liqueurs consumption, older women with BMI ≥25 kg/m^2^ s were more likely to belong to the trajectory of light consumption. Previous research indicated that adults who are physically less active were more likely to follow heavy drinking trajectories (Halonen et al. 2017, Sidorchuk et al. 2022). Our study found that young women engaging in moderate to high levels of physical activity were less likely to belong to the drinking trajectories of total alcohol, beer, and spirits/liqueurs, although this pattern was not appreciably observed for the wine drinking trajectories. Overall, membership in the wine light consumption trajectories was associated with more favorable profile, compared to the similar level of alcohol consumption from other beverages. This was reflected by the stronger positive association with high education levels and excellent SHR, weaker positive association with smoking, and stronger inverse association with a BMI ≥25 kg/m^2^, particularly observed among younger women.

### Limitations, methodological considerations, and future research

This study should be interpreted within the framework of the following limitations. The GBTM technique operates on the assumption that individual trajectories are homogeneous within the groups of trajectories. We acknowledge that there are unaccounted variations in individual trajectories, especially in smaller trajectory groups such as high to moderate or moderate to high. However, our analyses of differences between trajectory groups yielded results that are consistent with previous studies. Some degrees of selection and attrition bias are likely present in this study. The response rate at enrolment was highest from the northern part of Norway (Eiliv et al. 2003). Younger women and those with higher education level were more likely to complete follow-up questionnaires (Lund et al. 2008). However, the NOWAC cohort overall is considered to be nationally representative of Norwegian female population (Eiliv et al. 2003). Measurement error for alcohol consumption is likely present in this due to the self-reported data. In a validation NOWAC study, consumption of alcohol measured by the FFQ was lower compared to the consumption measured by 24-hour dietary recalls (Hjartåker et al. 2007). The Spearman’s rank correlation coefficient was .67 and the calibration coefficient was 1.47. Between the two methods, only 1%–2% of the women in this study were noted to be differently classified with regard to major dietary groups, including alcohol (Hjartåker et al. 2007). Another limitation is that the alcohol data collection questions used in NOWAC does not allow us to distinguish between lifetime or current abstainers. Alcohol data in this study collected from 1991 to 2011 may not reflect the current alcohol consumption patterns of Norwegian women.

Between younger and older women, we observed that the decreasing trajectory was more pronounced among younger women and the increasing trajectory was more pronounced among older women. Across these two age strata (31–49 and 50–70 years); we did not find many differences regarding the relationship between education, health status and lifestyle, and the trajectories of alcohol consumption. One difference was the relationship between higher education and the drinking trajectories of spirits/liqueurs observed among older but not younger women. These differences between younger and older women may be a combination of age and cohort effects, factors to be considered in alcohol-related research as predictive of alcohol use and of alcohol-related harms (Slade et al. 2016). Another methodological consideration for future studies within NOWAC cohort or beyond would be the consideration of abstainer’s bias arising from the choice of non-drinkers as the comparative group in alcohol-related health outcome observational studies (Rehm et al. 2008). In this study, women with poor health status were more likely to be non-drinkers. We did not test any specific hypothesis for why alcohol consumption changes during life. Further research on how life events (e.g. retirement), or health status changes influence alcohol consumption would help to better understand the increasing trends of alcohol consumption especially among older adults. Last, we could only address the level of drinking in this study. Other dimensions of alcohol consumption such as alcohol use disorder or binge drinking are of interested to be measured and analyzed by future studies.

## Conclusion

In this cohort of Norwegian women, we identified stable and unstable trajectories of alcohol consumption. The proportion of women in our study (around 16.5%) who remained in light drinking trajectories (5–10 g/day) it is a public health issues to be considered as 40% of the cancer cases in European women in 2017 were attributed to light drinking level (Rovira and Rehm 2021). Around 2.7% of women showed increases trends of alcohol consumption and these women were more likely to smoke, to report better health status, higher education, and a BMI below 25 kg/m^2^. Age around retirement (62–65 years) appeared to be a life period during which women could particularly increase their drinking. The increases trends of total alcohol consumption in this study were mirrored by increases in wine consumption, trend that is predicted to continue in Norway (Gustavsen and Rickertsen 2018). These findings provide information on groups and alcoholic beverages that can be targeted in alcohol-reduction polices and interventions.

## Supplementary Material

Proof_read_Supplementary_Material_ID_ALC-24-0133_agaf005

## Data Availability

The dataset used in this article cannot be shared publicly due to Norwegian ethical and security policies.
