# Supplementary material for: Alcohol consumption trajectories and associated factors in adult women: the Norwegian Women and Cancer study
Source: Alcohol Alcohol. 2025 Feb 8;60(2):agaf005. doi: 10.1093/alcalc/agaf005 (PMC11806201; doi:10.1093/alcalc/agaf005)
Supplement: Proof_read_Supplementary_Material_ID_ALC-24-0133_agaf005 [file proof_read_supplementary_material_id_alc-24-0133_agaf005.docx]

**Supplementary data content**

[Supplementary Panel 1 Literature review and gaps analysis 3](#_Toc188351802)

[Supplementary Table 1 Main features from the selected studies which sought to describe and to identify predictors of the longitudinal patterns of alcohol consumption in adults 5](#_Toc188351803)

[Supplementary Figure 1 Flowchart of study participants in the Norwegian Women and Cancer Study 1991-2011 7](#_Toc188351804)

[Supplementary Methods 1 Identification of classes of long-term alcohol consumption trajectories 8](#_Toc188351805)

[Supplementary Figure 2 Individual trajectories of total alcohol consumption (g/day) by each trajectory class in women aged 31-49 years at enrolment. The Norwegian Women and Cancer Study 1991-2011 14](#_Toc188351806)

[Supplementary Figure 3 Individual trajectories of alcohol consumption (g/day) from wine by each trajectory class in women aged 31-49 years at enrolment. The Norwegian Women and Cancer Study 1991-2011 15](#_Toc188351807)

[Supplementary Figure 4 Individual trajectories of alcohol consumption (g/day) from beer by each trajectory class in women aged 31-49 years at enrolment. The Norwegian Women and Cancer Study 1991-2011 16](#_Toc188351808)

[Supplementary Figure 5 Individual trajectories of alcohol consumption (g/day) from spirits/liqueurs by each trajectory class in women aged 31-49 years at enrolment. The Norwegian Women and Cancer Study 1991-2011 17](#_Toc188351809)

[Supplementary Figure 6 Individual trajectories of total alcohol consumption (g/day) by trajectory latent class in women aged 50-70 years at enrolment. The Norwegian Women and Cancer Study 1991-2011 18](#_Toc188351810)

[Supplementary Figure 7 Individual trajectories of alcohol consumption (g/day) from wine by each trajectory class in women aged 50-70 years at enrolment. The Norwegian Women and Cancer Study 1991-2011 19](#_Toc188351811)

[Supplementary Figure 8 Individual trajectories of alcohol consumption (g/day) from beer by each trajectory class in women aged 50-70 years at enrolment. The Norwegian Women and Cancer Study 1991-2011 20](#_Toc188351812)

[Supplementary Figure 9 Individual trajectories of alcohol consumption (g/day) from spirits/liqueurs by each trajectory class in women aged 50-70 years at enrolment. The Norwegian Women and Cancer Study 1991-2011 21](#_Toc188351813)

[Supplementary Figure 10 The odds ratios with 95% confidence interval of the adjusted associations between enrolment characteristics and wine trajectories in women aged 31-49 years at enrolment. The Norwegian Women and Cancer Study 1991-2011 22](#_Toc188351814)

[Supplementary Figure 11 The odds ratios with 95% confidence interval of the adjusted associations between enrolment characteristics and beer trajectories in women aged 31-49 years at enrolment. The Norwegian Women and Cancer Study 1991-2011 23](#_Toc188351815)

[Supplementary Figure 12 The odds ratios with 95% confidence interval of the adjusted associations between enrolment characteristics and spirits/liqueurs trajectories in women aged 31-49 years at enrolment. The Norwegian Women and Cancer Study 1991-2011 24](#_Toc188351816)

[Supplementary Figure 13 The odds ratios with 95% confidence interval of the adjusted associations between enrolment characteristics and wine trajectories in women aged 50-70 years at enrolment. The Norwegian Women and Cancer Study 1991-2011 25](#_Toc188351817)

[Supplementary Figure 14 The odds ratios with 95% confidence interval of the adjusted associations between enrolment characteristics and beer trajectories in women aged 50-70 years at enrolment. The Norwegian Women and Cancer Study 1991-2011 26](#_Toc188351818)

[Supplementary Figure 15 The odds ratios with 95% confidence interval of the adjusted associations between enrolment characteristics and spirits/liqueurs trajectories in women aged 50-70 years at enrolment. The Norwegian Women and Cancer Study 1991-2011 27](#_Toc188351819)

[Supplementary Table 3 Enrolment characteristics of the 16 women with alcohol intake higher than 100 g/day who excluded from this study 28](#_Toc188351820)

[Supplementary Table 4 Characteristics of women aged 31-49 years at enrolment according to trajectories of total alcohol consumption 29](#_Toc188351821)

[Abbreviation: SD, standard deviation; BMI, body mass index. 30](#_Toc188351822)

[Supplementary Table 5 Characteristics of women aged 50-70 years at enrolment according to trajectories of total alcohol consumption 31](#_Toc188351823)

[Supplementary Table 5 Continued 32](#_Toc188351824)

[Supplementary Figure 16 Mean predicted trajectories with 95% confidence intervals (dash lines) of total alcohol consumption (g/day) when excluding women who died before returning the third questionnaire. The Norwegian Women and Cancer Study 1991-2011 33](#_Toc188351825)

[Supplementary Figure 17 Mean predicted trajectories with 95% confidence intervals (dash lines) of total alcohol consumption (g/day) in women aged 31-49 years at enrolment when excluding women for whom the recall period of alcohol data assessment matched with periods of pregnancy. The Norwegian Women and Cancer Study 1991-2011 34](#_Toc188351826)

[Supplementary Figure 18 Mean trajectories with 95% confidence intervals (dash lines) of total alcohol consumption (g/day) and of alcohol consumption from spirits when removing the consumption data for liqueurs. The Norwegian Women and Cancer Study 1991-2011 35](#_Toc188351827)

[Supplementary references 37](#_Toc188351828)

[Stata codes of the final GBTM models 38](#_Toc188351829)

Supplementary Panel 1 Literature review and gaps analysis

Literature review

We systematically searched PubMed for studies published up to December 31st, 2022, using the search term: (("alcohol" OR "ethanol") AND ("trajectory" OR "trajectories" OR "long-term" OR "life course") AND ("predict" OR "determine" OR "determinant" OR "factors") AND ("cohort" OR "longitudinal studies")). The search was limited to observational studies, systematic reviews, and humans, yielding 73 papers. Seven additional papers were identified through reference list reviews. After reviewing titles and abstracts, we conducted full-text reviews and data extraction on 13 papers that aimed to describe the longitudinal patterns of alcohol consumption throughout adulthood and to examine factors (beyond age and gender) influencing the consumption (Supplementary Table 1). Exclusion criteria were studies focused on clinical samples, critical life periods (e.g., transition from adolescence to early adulthood, pregnancy, or pre and -post natal periods), and the change of alcohol consumption between two time-points. Eight out of 13 studies included only adults over 50 years old. The main statistical analysis methods used in these studies to compute longitudinal patterns of alcohol consumption were multilevel models (n=4), latent growth modelling (n=3), latent class growth modelling (n=4), and other (n=2). From the nine studies that modelled the trajectories of alcohol consumption, one study included men and only two reported gender-specific trajectory analysis. The two last studies used latent growth modelling, and none used latent class growth modelling to address the diversity of drinking over time in the population. Only one study extended the trajectory analysis to alcohol-specific drink consumption (wine and beer) and the trajectories were computed based on the pre-specification of alcohol consumption into three categories. Overall, previous studies focused on sociodemographic factors and health status, with few considering body mass index (BMI) (n=3) and physical activity level (n=3) as potential factors influencing longitudinal patterns of alcohol consumption.

**Box A** Gaps in the literature

| **Research gap type** |  |
| --- | --- |
| Evidence gap | 1. While most women sustain their light drinking levels, some can increase or decrease it during adulthood. Evidence using repeated individual measurements is limited. 2. Obesity and physical activity are potential factors associated with longitudinal alcohol consumption, but the evidence is contradictory and limited. 3. The association between smoking and spirits, beer consumption is stronger compared to wine, but the evidence is limited. |
| Knowledge gap | 1. Do long-term light drinkers have more favourable profile than long-term non-drinkers? 2. Do women sustain, increase or decrease wine, beer, spirits or liqueurs consumption during adulthood? What are the factors that drive these trends? 3. Do long-term wine drinkers have more favourable profile than the long-term beer or spirit/liquor drinkers? |
| Methodological gap | 1. Small sample size of previous longitudinal studies. 2. Adults over 50 years old were mainly included in previous longitudinal studies. 3. Recall bias in several studies which used retrospective alcohol data collection (recall of past drinking levels). |
| Theoretical gap | 1. At what age are adult women more likely to experience increases of alcohol consumption? |
| Population gap | 1. Women. |

Supplementary Table 1 Main features from the selected studies which sought to describe and to identify predictors of the longitudinal patterns of alcohol consumption in adults

| **Reference in the main manuscript, country** | **Follow-up period**  **(length in years)** | **Number of surveys** | **Sample size**  **(% of men)** | **Age at enrolment** | **Computation of alcohol consumption dimensions** | **Main statistical analysis used to compute the longitudinal patterns of alcohol consumption** |
| --- | --- | --- | --- | --- | --- | --- |
| Brennan 2011^a^,  US | NA  (20) | 5 | 719  (55.5) | 55-65 (range) | Quantity: drinks per day.  Drinking problems defined by Drinking Problem Index | Latent growth modelling to identify trajectories of alcohol consumption quantity and drinking problems. |
| Holton 2019^a^,  Ireland | 2009-2015  (4) | 3 | 4 295  (45) | ≥50 | Quantity: average drinks/week.  Frequency: non-drinkers; monthly/special occasion; weekly infrequent; weekly frequent.  Pattern: non-drinkers; moderate (1-5 drinks/occasion); heavy episodic (≥6 drinks/occasion).  1 standard drink = 10 g of ethanol. | Mixed models to assess the changes in frequency, quantity, and patterns of alcohol consumption in relation to participant characteristics. |
| McEvoy 2013^a^,  US | 1984-2009  (15) | 2-6 | 1 076  (42.9) | 66.4 (mean)  50-89 (range) | Quantity: g/week. | Mixed models to assess changes in quantity alcohol consumption in relation to health status. |
| Skourlis 2021 ^a,b^,  Greece | 1994-2011  (17) | 3-4 | 22 721  (41.8) | 53 (mean) | Quantity for total alcohol: g/week.  Quantity for alcohol-specific drinks: glasses/week. | Mixed models to assess changes in the quantity of total alcohol and alcohol-specific drinks consumption in relation to participant characteristics. |
| Moore 2005,  US | 1971-1992  (20) | 1-4 | 14 105  (41.8) | 25-74 (range) | Quantity: drinks/week. | Mixed models to assess changes in the quantity of alcohol consumption in relation to participant characteristics. |
| Agahi 2022,  Finland | 2014-2019  (5) | 5 | 1 393  (17) | ≥60 | Heavy drinking pattern:  exceeding 14 units/week.  1 standard drink = 12 g of ethanol. | Latent class growth modelling to identify the trajectories of heavy alcohol drinking. |
| Agahi 2022 ^a^,  Sweden | 2015-2018  (3) | 1-4 | 3 335  (47.3) | 60-66  (range) | Quantity: units per week. | Latent growth modelling to identify trajectories of alcohol consumption quantity over the retirement transition. |
| Halonen 2017,  Finland | 2002-2013  (4-12) | 2-6 | 5 805  (20) | ≥58 | Risky drinking pattern:  exceeding 24 units/week for men and 16 for women.  1 standard unit = 12 g of ethanol. | Latent class growth modelling to identify trajectories of risky alcohol drinking over the retirement transition. |
| Platt 2011,  US | 1992-2006  (14) | 5-8 | 6 787  (45) | 51-61 (range) | Quantity: drinks/day. | Alcohol consumption trajectories computed based on the alcohol quantity reported in each survey. |
| Bobo 2013,  US | 1998-2008  (10) | 3-6 | 5 805  (100) | 50-65 (range) | Quantity: drinks/day. | Latent class growth modelling to identify the trajectories of alcohol consumption quantity. |
| Sidorchuk 2022^b^,  Sweden | 2002-2014  (12) | 3 | 13 152  (43) | 49.2 (mean)  25-84 (range) | Patterns: Non-drinkers; moderate (>0 to ≤168 g/week for women and >0 to ≤252 g/week for men); heavy (>168 g/week for women and > 252 g/week for men). | Alcohol consumption trajectories computed based on the pre-specification of alcohol consumption patterns in each survey. |
| Baumann 2022,  Germany | 2018-2022  (3) | 4 | 1 638  (44) | 31 (mean)  18-64 (range) | Patterns: at-risk (AUDIT score 4-12 for women and 5-12 for men); low risk (AUDIT score 1-3 for women and 1-4 for men). | Latent class growth modelling to identify the trajectories of AUDIT score among at-risk drinkers and low-risk drinkers. |
| Dobson 2018,  Canada | 1994-2010  (16) | 9 | 5 458  (52.5) | 37 (mean) | Quantity: drinks per day. | Latent class growth modelling to identify the trajectories of alcohol consumption quantity. |
| **Notes:** ^a^ Reported gender-strata analysis of longitudinal alcohol consumption; ^b^ Reported analysis for beverage-specific analysis.  **Abbreviations:** NA, not available; AUDIT, Alcohol Use Disorders Identification Test. | | | | | | |

Supplementary Figure 1 Flowchart of study participants in the Norwegian Women and Cancer Study 1991-2011

**N = 102 392**

Enrolled from 1991 to 1997

**21 587**

Completed only one questionnaire

**N = 80 805**

**4 407**

Had incomplete data on alcohol

**N = 76 398**

**16**

Extreme alcohol consumption (>100 g/day)

**N = 76 382**

**58 564** Eligible for trajectory analyses in women aged 31-49 years at enrolment

**17 818** Eligible for trajectory analyses in women 50-70 years at enrolment

Supplementary Methods 1 Identification of classes of long-term alcohol consumption trajectories

The trajectories were modelled using group-based trajectory modelling (GBTM) separately in two subcohorts defined by age at enrolment, 31-49, and 50-70 years. This grouping was determined after observing similar trajectories of alcohol consumption between the age groups 31-39 and 40-49 years. To ensure an adequate sample size, the age groups 50-59 years and 60-70 years, representing respectively 15% (n=11456) and 8% (n=6362) of the study sample, were merged.

Following Andruff and colleagues,^1^ the process of choosing polynomial order started with the maximum order (quadratic).

For our data set with three time points, a single quadratic trajectory model was tested first. Once the quadratic order was significant (*P*-value ≤0.05), the next quadratic order was tested. If non-significance was shown in one or all polynomial orders, the lower (linear or intercept) order was tested. We used the Bayesian information criterion (BIC) as a fit index to compare competing models with different number of trajectories ^2,3^. The log Bayes Factor was calculated to measure the extent of evidence surrounding the model with the highest number of trajectories. The average posterior probability of assignment for each class of 0.7 or higher, a close correspondence between the proportion of a sample assigned to a certain class and the proportion estimated from the model (Table A, Table B below), and a visually reasonably narrow confidence interval (CI) around each trajectory were considered for evaluating the model accuracy ^2,3^. To assess the relevance of trajectories, the individual trajectories of alcohol consumption for each trajectory class were plotted (Supplementary Figures 2-9 below). Here, the point was to ensure that the trajectory classes were realistic and meaningful. This means that the estimated trajectory classes should be visibly distinct and consistent with respect to the observed individual trajectories. To improve the convergence of the model, women in the subcohort 31-49 years with a higher consumption than 100 g/day of total alcohol (n=16), higher than 60 g/day of wine (n=3), and higher than 60 g/day of spirits and liqueurs (n=2) at enrollment were excluded when modelling the trajectories of total alcohol, wine and spirits and liqueurs consumption. For the same reason, one woman in the subcohort 50-70 years with a higher consumption than 20 g/day of beer reported at the third questionnaire was excluded when modelling the trajectories of beer consumption.

Additionally, an alternative approach was applied for GBTM selection which consists of starting the model with a zero-component polynomial for the participants with no or very low consumption of alcohol over time, followed by the approach proposed by Andruff and colleagues,^1^ as described above. The alternative approach has also been used in previous studies analysing the trajectories of alcohol consumption.^4^ We observed improvement of discrimination ability of GBTM model when analysing the trajectories of total alcohol consumption in the subcohort 31-49 years. Using the alternative approach, the GBTM model group showed closer correspondence for the non-drinker stable trajectory between the proportion of the sample assigned based on the posterior probability and the proportion estimated from the model. Furthermore, when using the alternative approach, the non-drinker stable trajectory was comprised by 94.3% of women who reported 0 g/day of total alcohol consumption in the three questionnaires and 5.7% by women who reported a maximum of 0.58 g/day total alcohol consumption during the study period. When using the approach proposed by Andruff and colleagues,^1^ the non-drinker stable trajectory was comprised by 66.7% of women who reported 0 g/day of total alcohol consumption in the three questionnaires and 33.3% by women who reported a maximum of 1.98 g/day total alcohol consumption during the study period. Therefore, the alternative approach was finally used when analysing the trajectories of total alcohol consumption in the subcohort 31-49 years.

**Supplementary Methods 1a** Trajectory models with different number of groups. Analysis for total alcohol consumption in women aged 31-49 years at enrolment


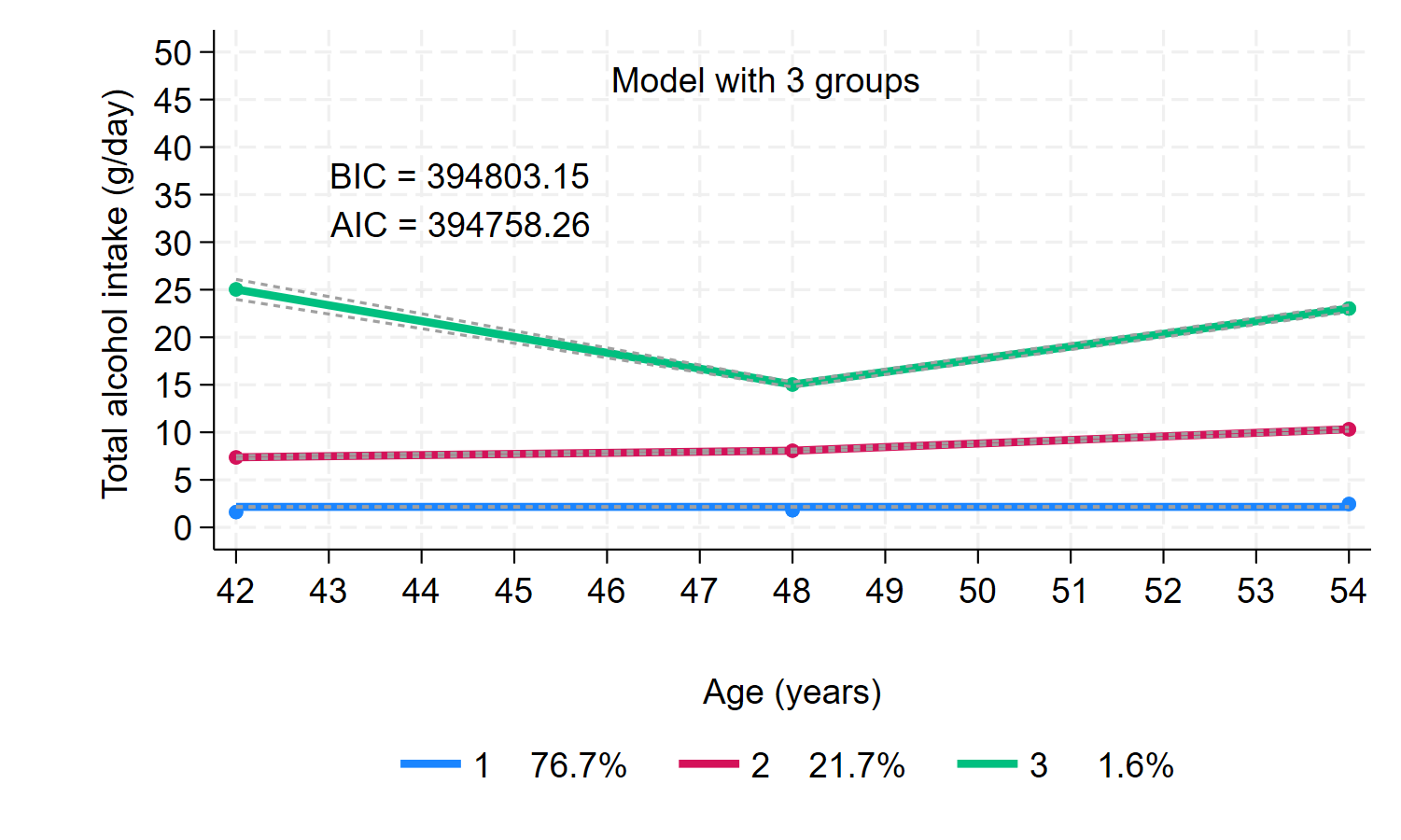


**
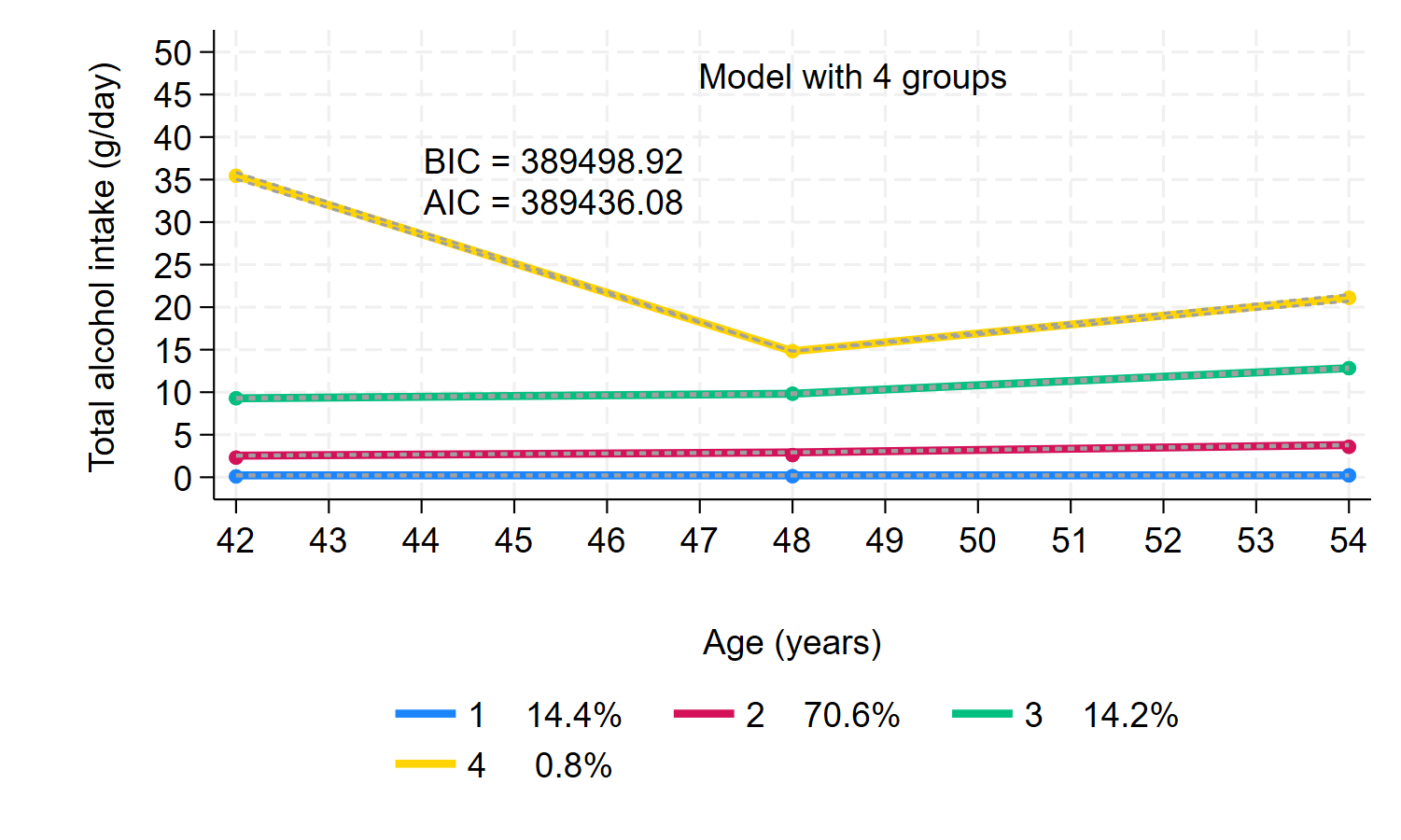
**

**
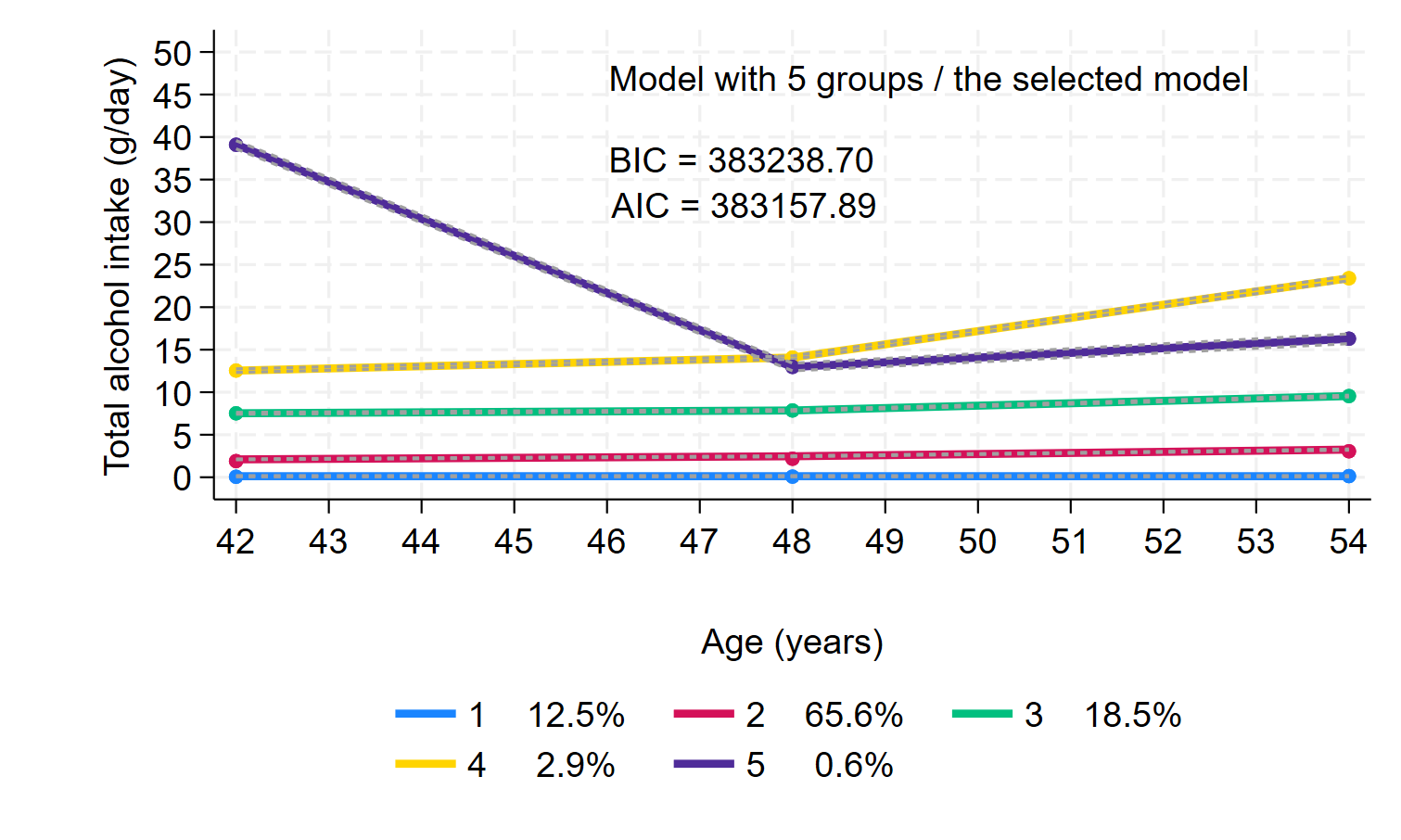
**

Notes: The average posterior probabilities in these models were higher than 0.70 in all groups.

**Supplementary Methods 1b** Trajectory models with different number of groups. Analysis for total alcohol consumption in women aged 50-70 years at enrolment


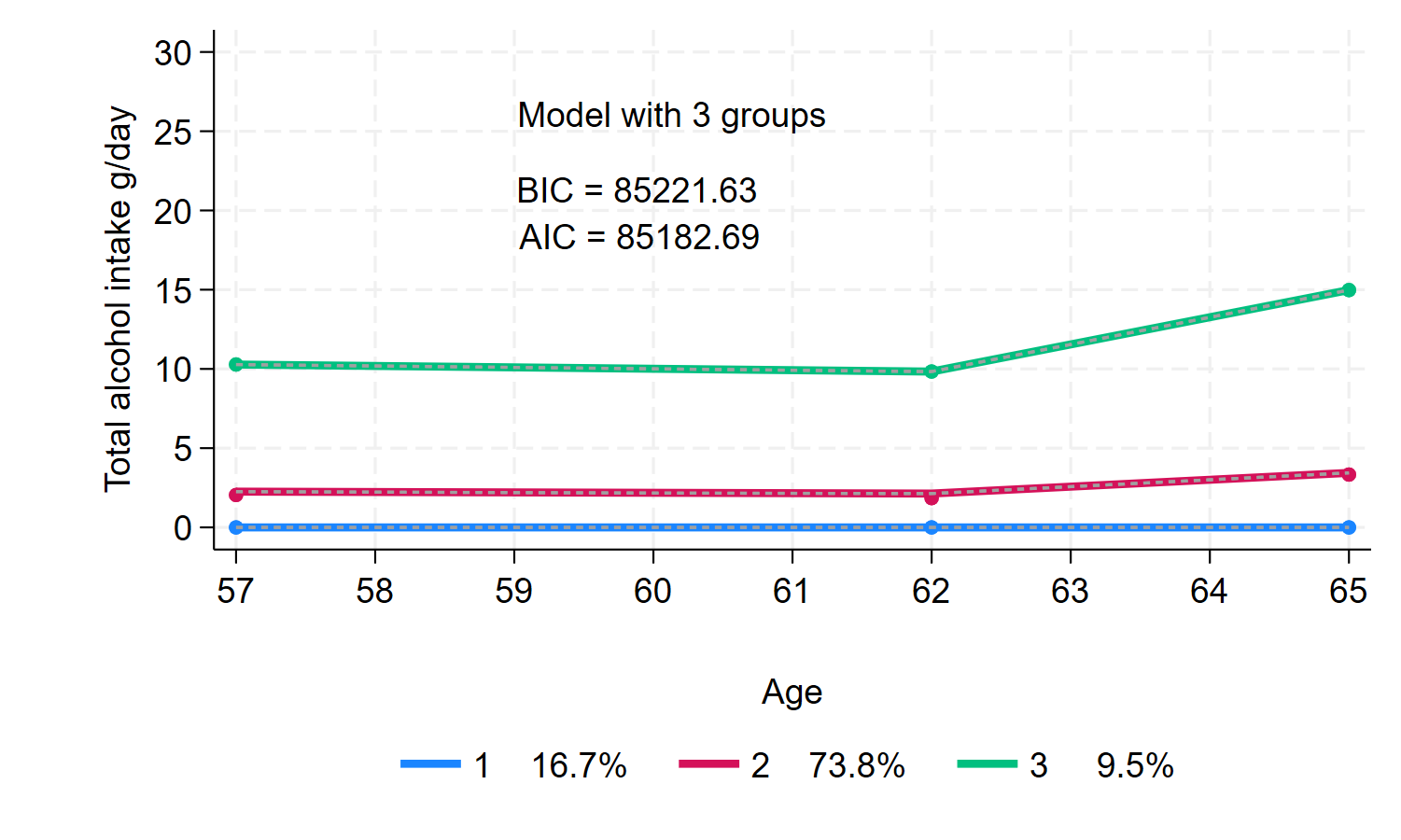


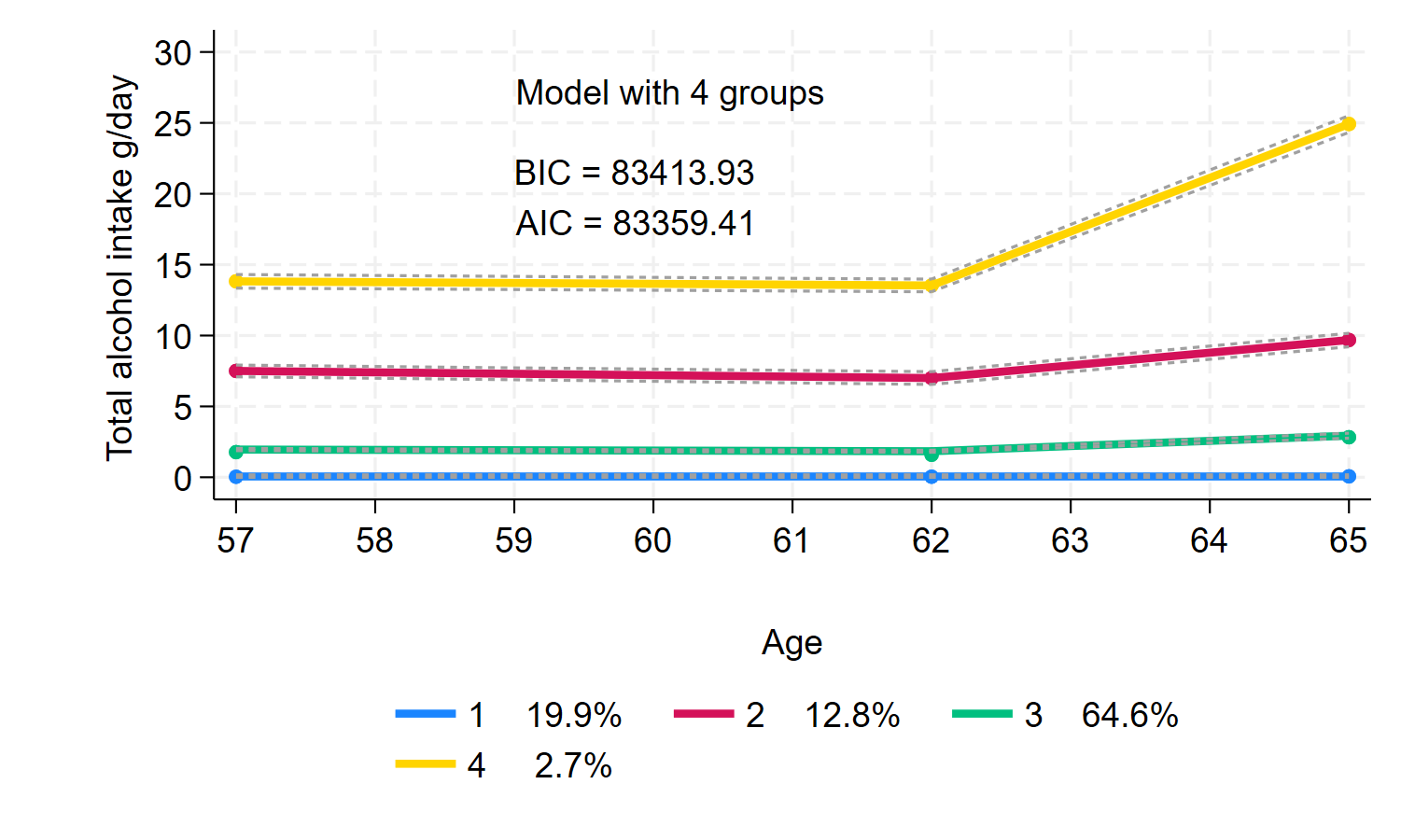


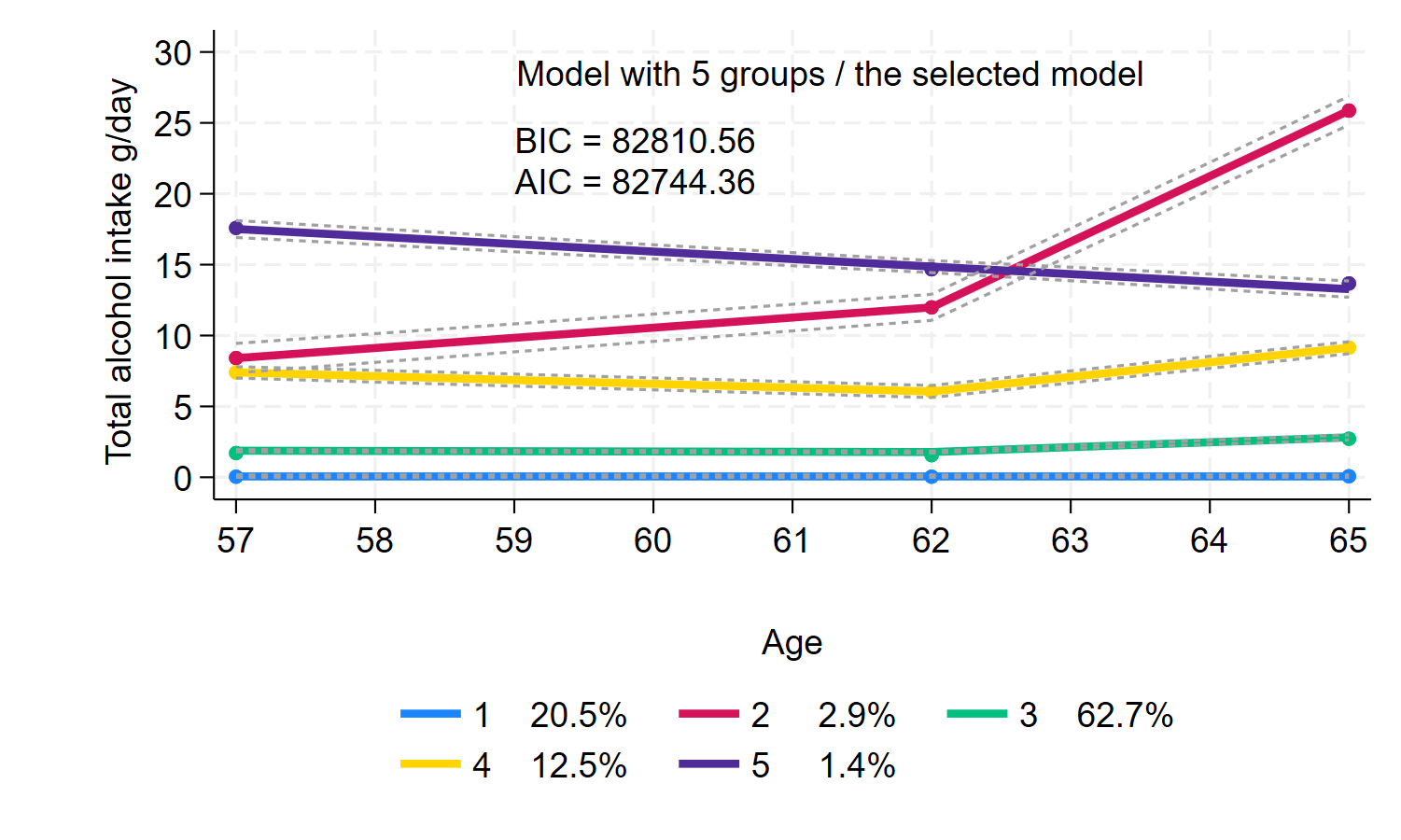


Notes: The average posterior probabilities in these models were higher than 0.70 in all groups.

**Table 2a** Estimated parameters of the final selected trajectory models in women aged 31-49 years at enrolment

| **Trajectories** | **Average posterior probability of assignment** | **Proportion (%) estimated by the model** | **Proportion (%) based on the assigned probability** |
| --- | --- | --- | --- |
| **Total alcohol consumption** | | | |
| Non-drinker stable | 0.79 | 12.5 | 12.5 |
| Low stable | 0.92 | 65.6 | 66.3 |
| Light increasing | 0.88 | 18.5 | 17.8 |
| Moderate to high | 0.92 | 2.9 | 2.8 |
| High to moderate | 0.98 | 0.6 | 0.6 |
| **Wine consumption** | | | |
| Non-drinker stable | 0.78 | 22.1 | 23.5 |
| Low stable | 0.89 | 59.8 | 60.7 |
| Light increasing | 0.94 | 16.3 | 14.1 |
| Moderate to high | 0.92 | 1.6 | 1.5 |
| High to moderate | 1.00 | 0.3 | 0.3 |
| **Beer consumption** | | | |
| Non-drinker stable | 0.85 | 37.5 | 40.6 |
| Low stable | 0.94 | 58.3 | 55.5 |
| Light stable | 0.95 | 4.1 | 3.8 |
| High to light | 0.99 | 0.1 | 0.1 |
| **Spirits/liqueurs consumption** | | | |
| Non-drinker stable | 0.86 | 55.5 | 57.9 |
| Low stable | 0.85 | 41.7 | 39.6 |
| Light to high | 0.95 | 2.8 | 2.5 |

**Table 2c** Estimated parameters of the final selected trajectory models in women aged 50-70 years at enrolment

| **Trajectories** | **Average posterior probability of assignment** | **Proportion (%) estimated by the model** | **Proportion (%) based on the assigned probability** |
| --- | --- | --- | --- |
| **Total alcohol consumption** | | | |
| Non-drinker stable | 0.78 | 20.5 | 23.6 |
| Low stable | 0.93 | 62.7 | 60.1 |
| Light unstable | 0.84 | 12.5 | 12.1 |
| Light to high | 0.82 | 2.9 | 2.7 |
| Moderate decreasing | 0.91 | 1.4 | 1.4 |
| **Wine consumption** | | | |
| Non-drinker stable | 0.70 | 22.6 | 29.9 |
| Low stable | 0.94 | 59.7 | 52.9 |
| Low to light | 0.88 | 11.8 | 11.4 |
| Low to high | 0.95 | 2.7 | 2.8 |
| Light stable | 0.94 | 3.1 | 3 |
| **Beer consumption** | | | |
| Non-drinker stable | 0.87 | 51.9 | 55 |
| Low stable | 0.90 | 45.4 | 42.2 |
| Light unstable | 0.91 | 2.7 | 2.8 |
| **Spirits/liqueurs consumption** | | | |
| Non-drinker stable | 0.85 | 62.4 | 68.2 |
| Low stable | 0.86 | 36.2 | 30.5 |
| Light to moderate | 0.99 | 1.4 | 1.4 |

Supplementary Figure 2 Individual trajectories of total alcohol consumption (g/day) by each trajectory class in women aged 31-49 years at enrolment. The Norwegian Women and Cancer Study 1991-2011


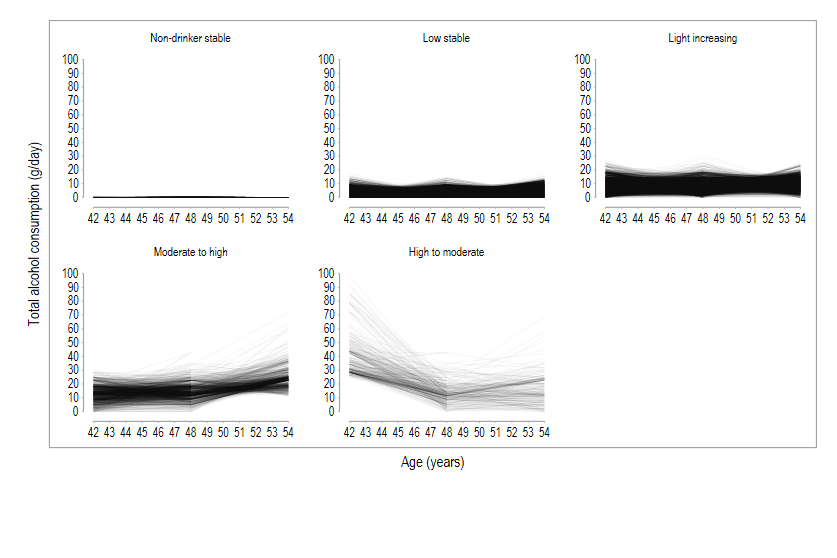


Supplementary Figure 3 Individual trajectories of alcohol consumption (g/day) from wine by each trajectory class in women aged 31-49 years at enrolment. The Norwegian Women and Cancer Study 1991-2011

**
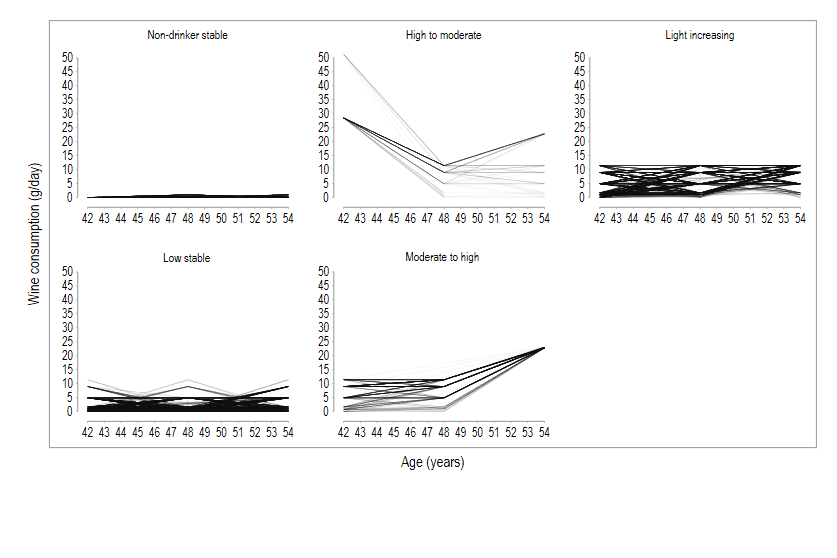
**

Supplementary Figure 4 Individual trajectories of alcohol consumption (g/day) from beer by each trajectory class in women aged 31-49 years at enrolment. The Norwegian Women and Cancer Study 1991-2011

**
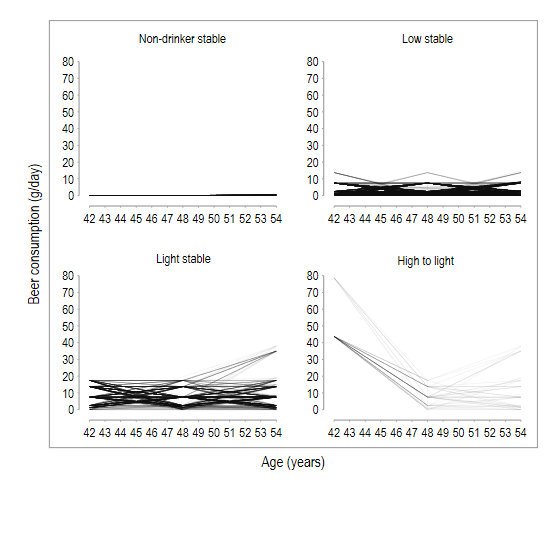
**

Supplementary Figure 5 Individual trajectories of alcohol consumption (g/day) from spirits/liqueurs by each trajectory class in women aged 31-49 years at enrolment. The Norwegian Women and Cancer Study 1991-2011

**
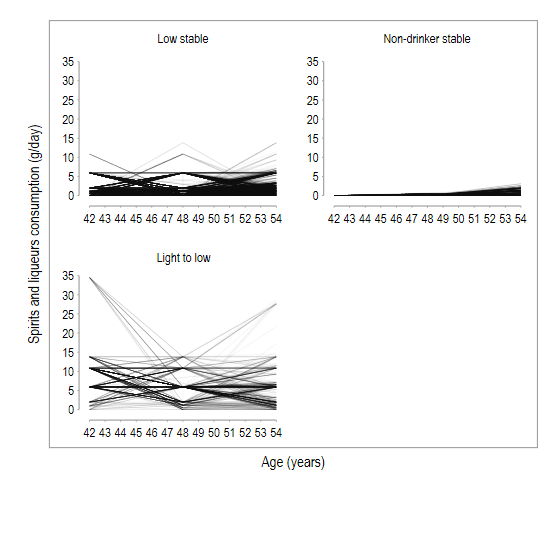
**

Supplementary Figure 6 Individual trajectories of total alcohol consumption (g/day) by trajectory latent class in women aged 50-70 years at enrolment. The Norwegian Women and Cancer Study 1991-2011

**
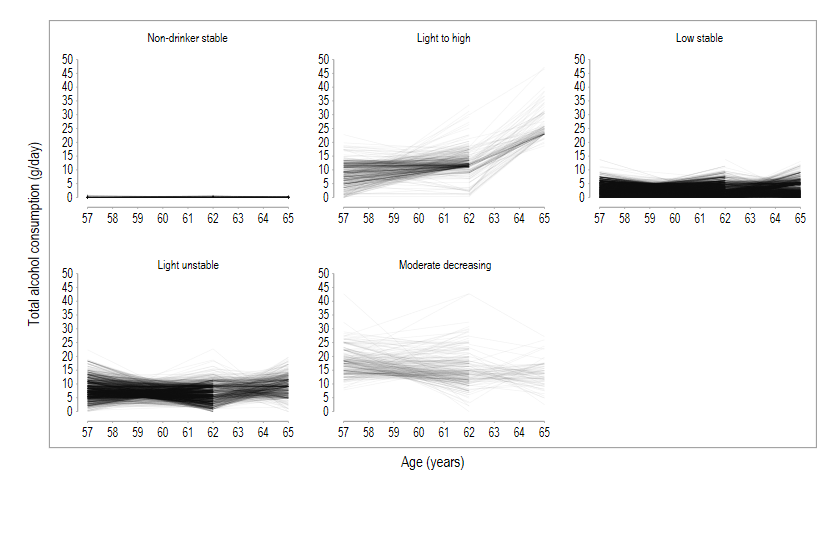
**

Supplementary Figure 7 Individual trajectories of alcohol consumption (g/day) from wine by each trajectory class in women aged 50-70 years at enrolment. The Norwegian Women and Cancer Study 1991-2011

**
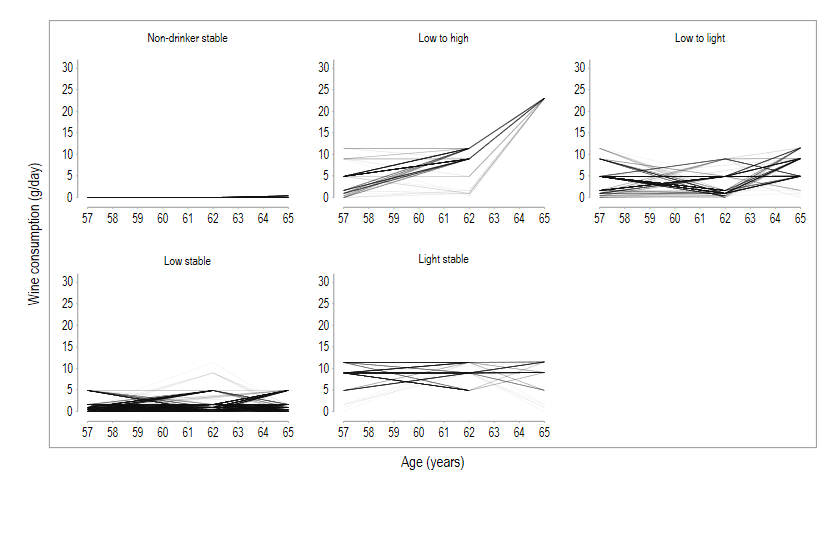
**

Supplementary Figure 8 Individual trajectories of alcohol consumption (g/day) from beer by each trajectory class in women aged 50-70 years at enrolment. The Norwegian Women and Cancer Study 1991-2011

**
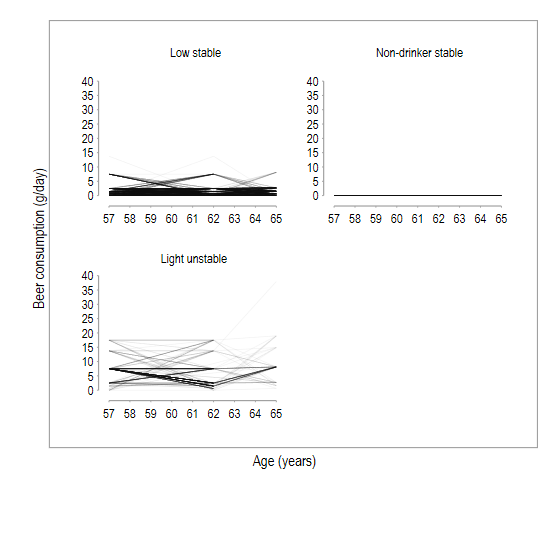
**

Supplementary Figure 9 Individual trajectories of alcohol consumption (g/day) from spirits/liqueurs by each trajectory class in women aged 50-70 years at enrolment. The Norwegian Women and Cancer Study 1991-2011

**
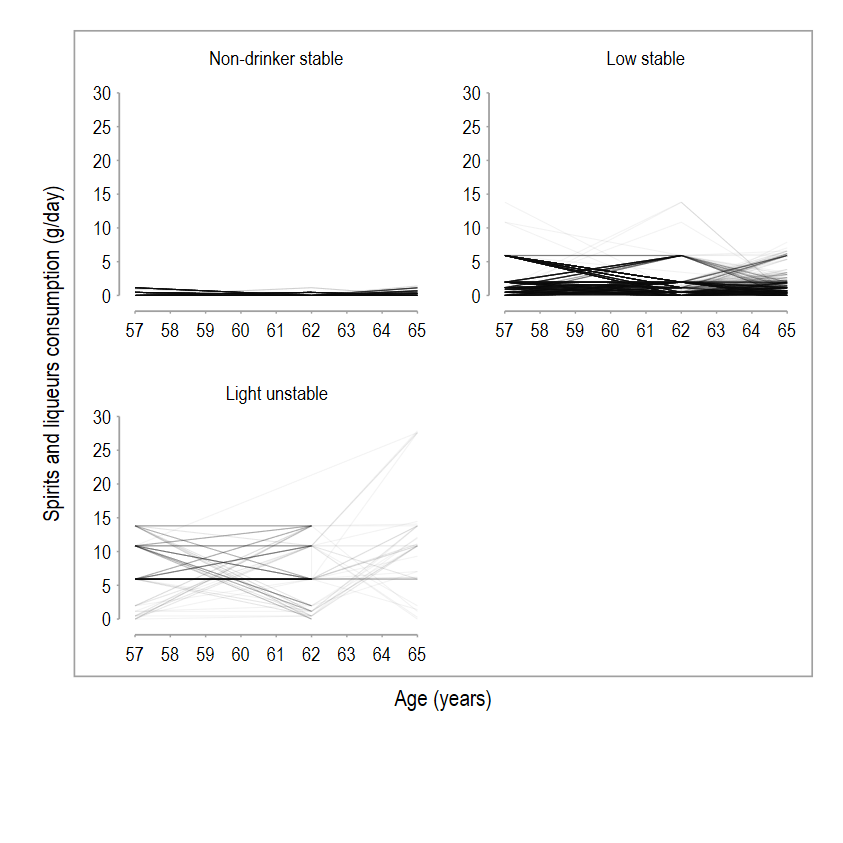
**

Supplementary Figure 10 The odds ratios with 95% confidence interval of the adjusted associations between enrolment characteristics and wine trajectories in women aged 31-49 years at enrolment. The Norwegian Women and Cancer Study 1991-2011

*Abbreviations: SRH, self-rated health; PA, physical activity level.*

The reference category for the dependent variable was low stable trajectory.

**
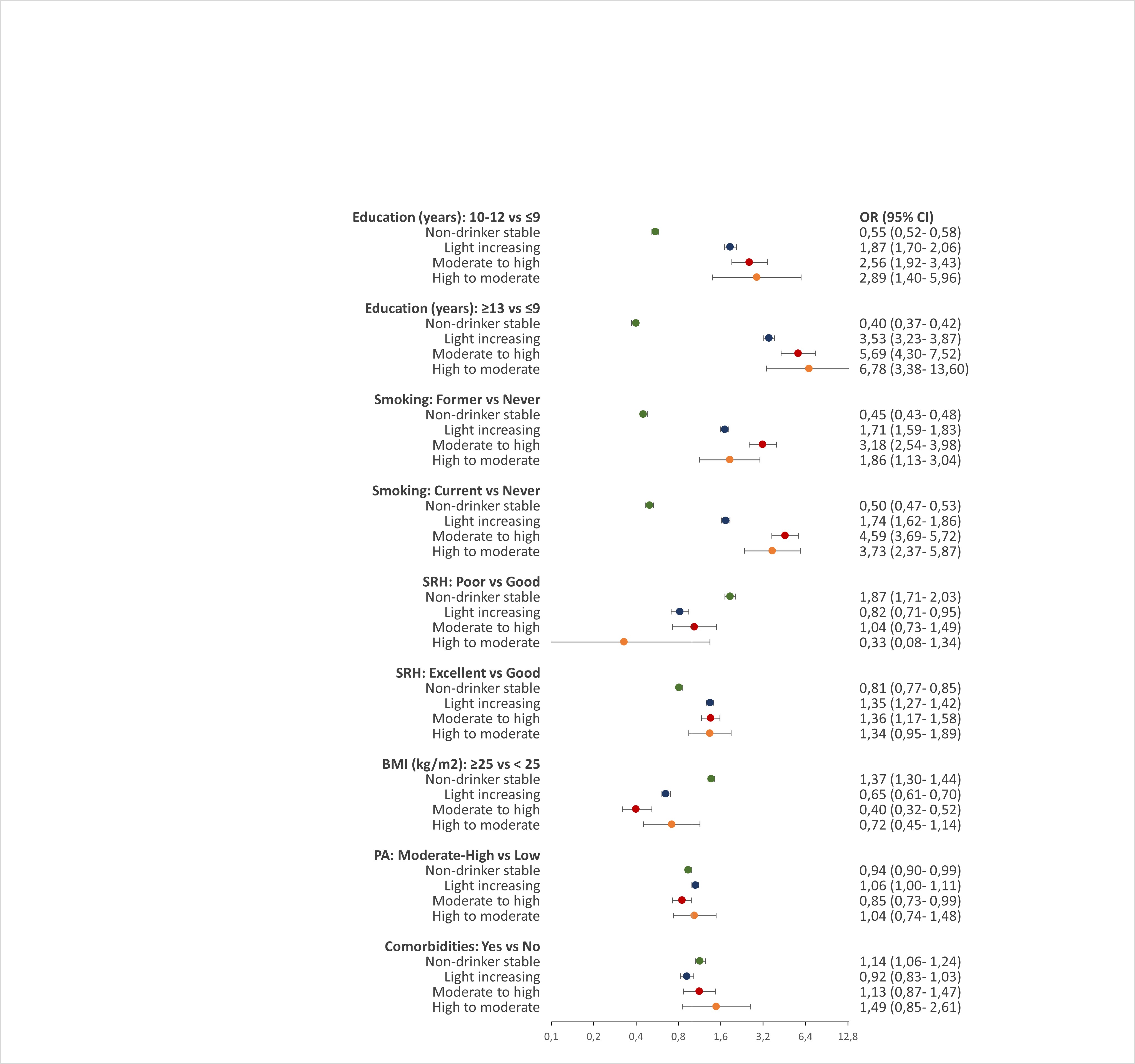
**

Supplementary Figure 11 The odds ratios with 95% confidence interval of the adjusted associations between enrolment characteristics and beer trajectories in women aged 31-49 years at enrolment. The Norwegian Women and Cancer Study 1991-2011

*Abbreviations: SRH, self-rated health; PA, physical activity level.*

The reference category for the dependent variable was low stable trajectory.

**
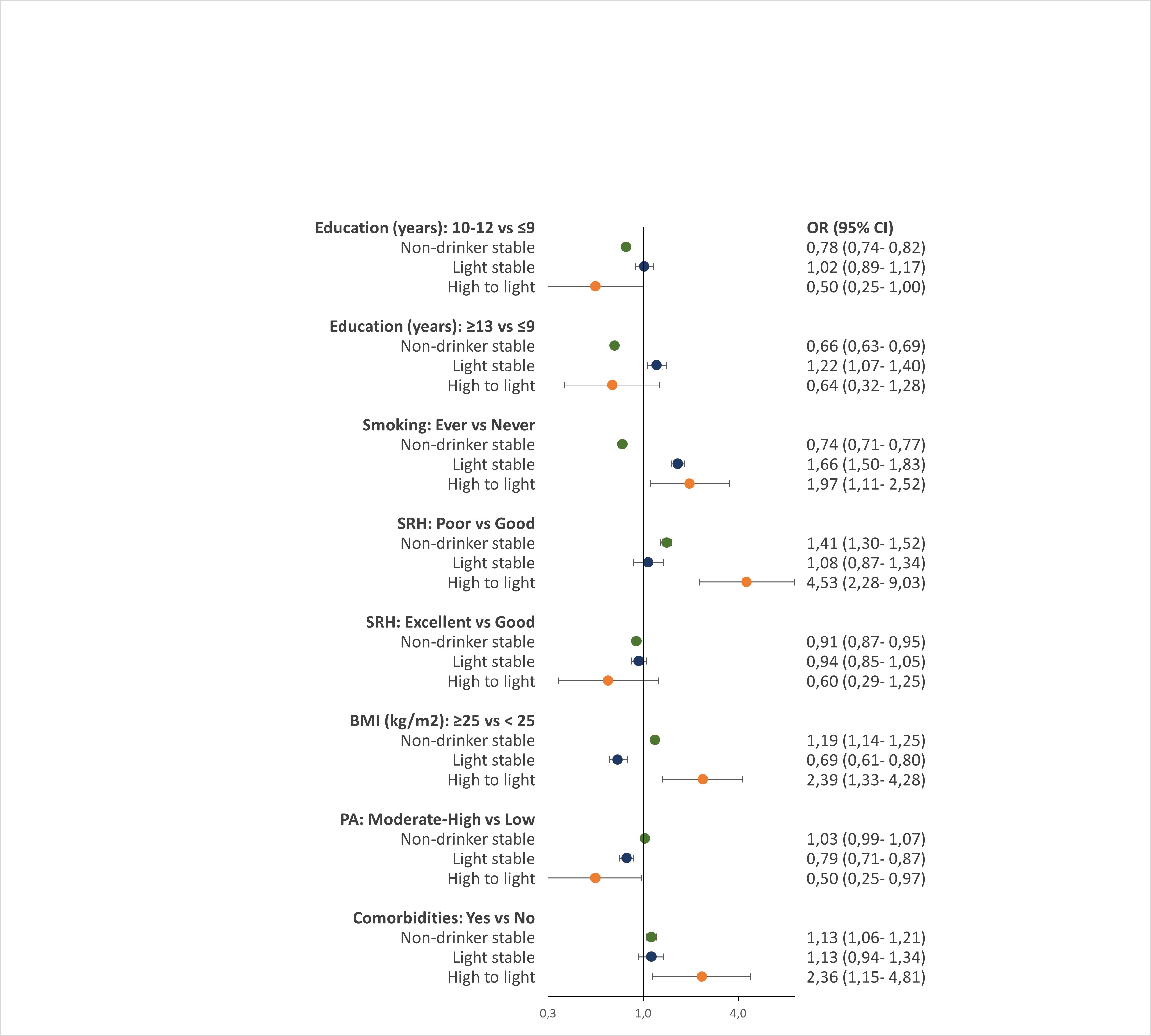
**

***** Because of lack of statical power, a binary variable of smoking status (never, ever smokers) was used when testing the association between smoking status and beer consumption trajectories.

Supplementary Figure 12 The odds ratios with 95% confidence interval of the adjusted associations between enrolment characteristics and spirits/liqueurs trajectories in women aged 31-49 years at enrolment. The Norwegian Women and Cancer Study 1991-2011

*Abbreviations: SRH, self-rated health; PA, physical activity level.*

The reference category for the dependent variable was low stable trajectory.


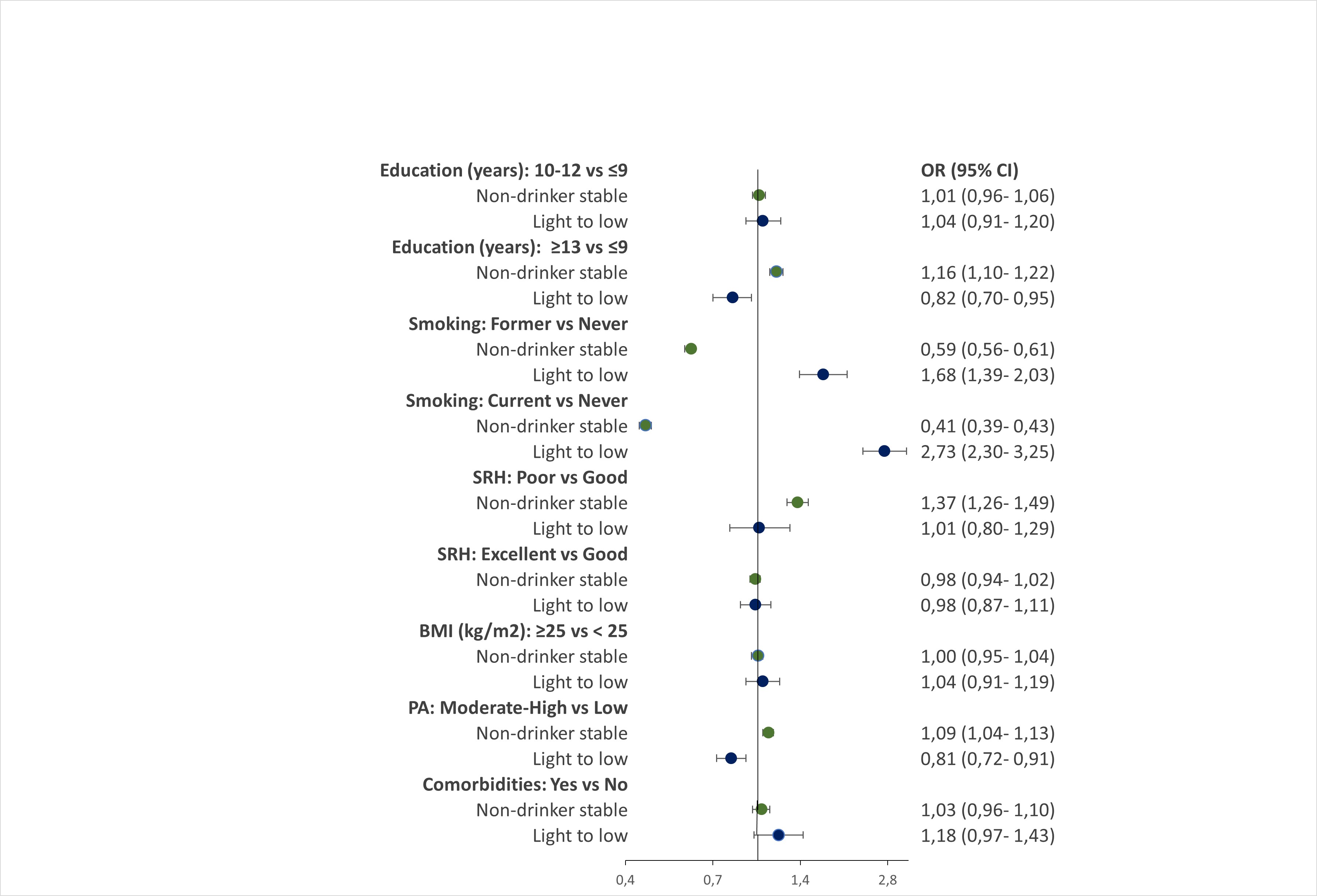


Supplementary Figure 13 The odds ratios with 95% confidence interval of the adjusted associations between enrolment characteristics and wine trajectories in women aged 50-70 years at enrolment. The Norwegian Women and Cancer Study 1991-2011

*Abbreviations: SRH, self-rated health; PA, physical activity level.*

The reference category for the dependent variable was low stable trajectory.

**
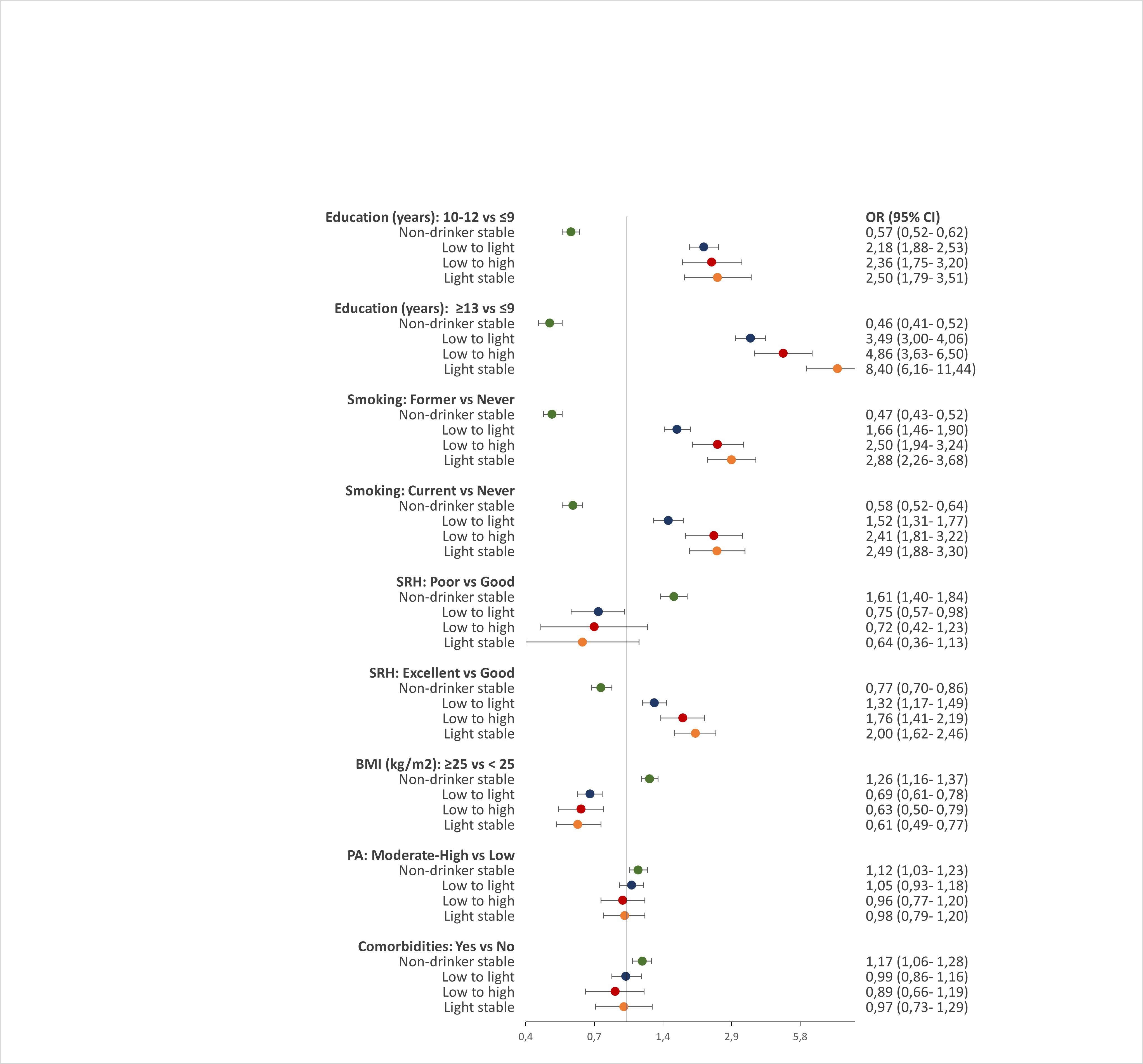
**

Supplementary Figure 14 The odds ratios with 95% confidence interval of the adjusted associations between enrolment characteristics and beer trajectories in women aged 50-70 years at enrolment. The Norwegian Women and Cancer Study 1991-2011

*Abbreviations: SRH, self-rated health; PA, physical activity level.*

The reference category for the dependent variable was low stable trajectory.

**
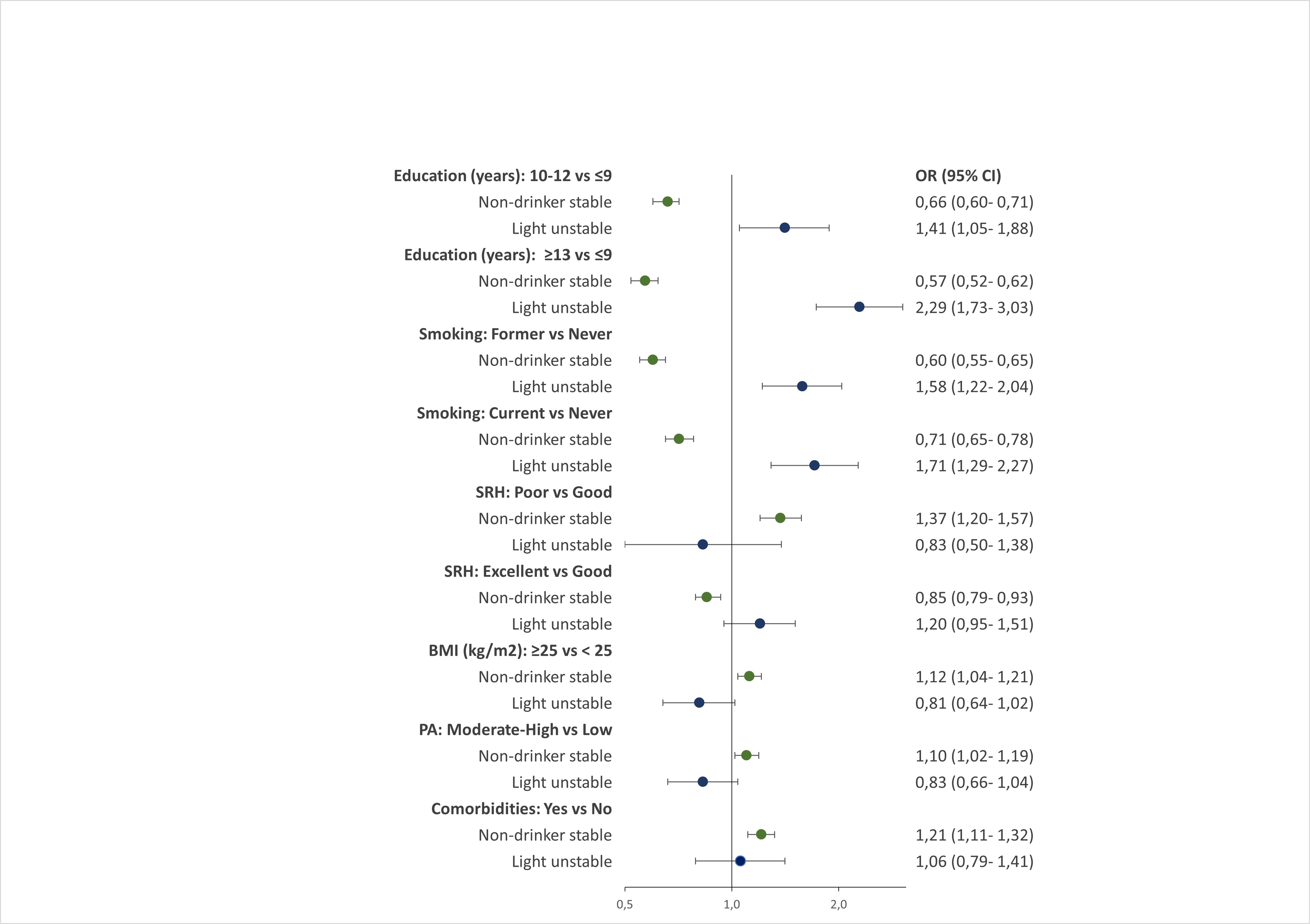
**

Supplementary Figure 15 The odds ratios with 95% confidence interval of the adjusted associations between enrolment characteristics and spirits/liqueurs trajectories in women aged 50-70 years at enrolment. The Norwegian Women and Cancer Study 1991-2011

*Abbreviations: SRH, self-rated health; PA, physical activity level.*

The reference category for the dependent variable was low stable trajectory.


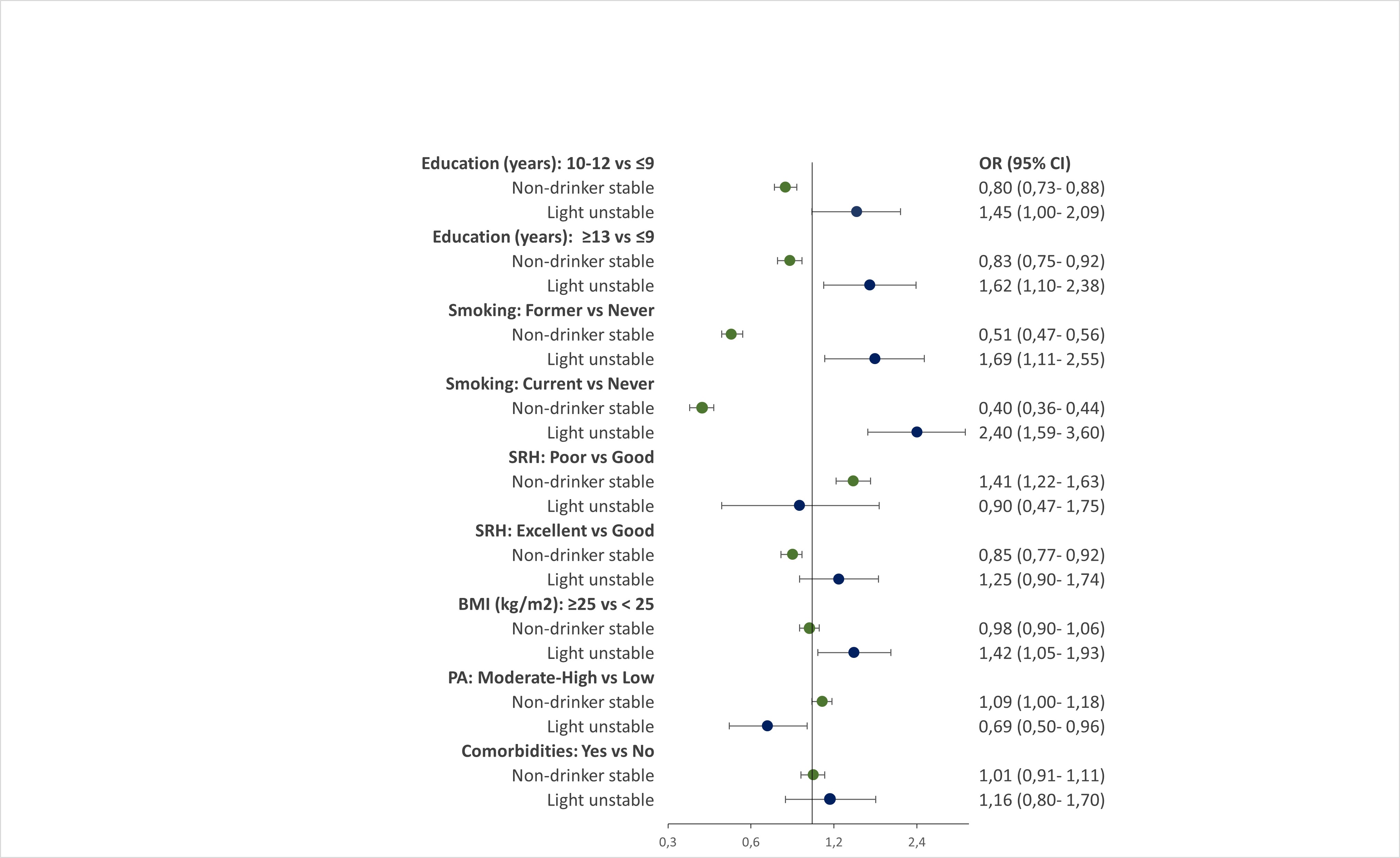


Supplementary Table 3 Enrolment characteristics of the 16 women with alcohol intake higher than 100 g/day who excluded from this study

| **Characteristics** | | **Number of women** |
| --- | --- | --- |
| Years of education | |  |
|  | ≤9 | 7 |
|  | 10-12 | 4 |
|  | ≥13 | 4 |
|  | Missing | 1 |
| Current smoker | |  |
|  | Yes | 5 |
|  | No | 8 |
|  | Missing | 3 |
| Self-rated health | |  |
|  | Poor | 4 |
|  | Good | 8 |
|  | Excellent | 3 |
|  | Missing | 2 |
| BMI categories | |  |
|  | <25 kg/m2 | 9 |
|  | ≥25 kg/m2 | 5 |
|  | Missing | 2 |
| Physical activity level | |  |
|  | Low | 6 |
|  | Moderate-High | 5 |
|  | Missing | 5 |
| History of comorbidities | |  |
|  | No | 14 |
|  | Yes | 2 |

Abbreviation: SD, standard deviation; IQR, interquartile range; BMI, body mass index.

Women who attended at least two surveys were included in this study.

Supplementary Table 4 Characteristics of women aged 31-49 years at enrolment according to trajectories of total alcohol consumption

|  | | **Non-drinker stable** | **Low stable** | **Light increasing** | **Moderate to high** | **High to moderate** |
| --- | --- | --- | --- | --- | --- | --- |
| No (%) of women | | 7 332 (12.5) | 38 835 (66.3) | 10 446 (17.8) | 1 610 (2.8) | 341 (0.6) |
| Age, mean (SD), years | | 42.1 (4.6) | 41.4 (4.6) | 41.6 (4.5) | 41.9 (4.5) | 42.1 (4.4) |
| Total alcohol in g/day at 1st survey, mean (SD) | | 0 | 1.9 (1.4) | 7.8 (3.9) | 12.7 (5.7) | 39.1 (14.3) |
| Alcohol from wine in g/day at 1st survey, mean (SD) | | 0 | 0.8 (0.9) | 3.6 (2.9) | 5.2 (3.8) | 17.4 (15.3) |
| Alcohol from beer in g/day at 1st survey, mean (SD) | | 0 | 0.7 (0.8) | 2.7 (2.3) | 4.8 (4.6) | 14.7 (13.8) |
| Alcohol from spirits/liqueurs in g/day at 1st survey, mean (SD) | | 0 | 0.4 (0.3) | 1.5 (1.3) | 2.7 (1.5) | 7 (6.8) |
| Total Alcohol in g/day at 2nd survey, mean (SD) | | 0 | 2.2 (1.9) | 8.1 (3.5) | 14.4 (5.6) | 12.9 (7.5) |
| Alcohol from wine in g/day at 2nd survey, mean (SD) | | 0 | 1.2 (0.9) | 4.6 (3.1) | 6.8 (3.6) | 6.1 (4.4) |
| Alcohol from beer in g/day at 2nd survey, mean (SD) | | 0 | 0.7 (0.6) | 2.3 (2.2) | 4.9 (4.8) | 4.4 (4.3) |
| Alcohol from spirits/liqueurs in g/day at 2nd survey, mean (SD) | | 0 | 0.3 (0.3) | 1.2 (1) | 2.7 (2.4) | 2.3 (2.1) |
| Total alcohol in g/day at 3rd survey, mean (SD) | | 0 | 3 (2.4) | 9.8 (3.5) | 23.6 (6.8) | 16.2 (10.8) |
| Alcohol from wine in g/day at 3rd survey, mean (SD) | | 0 | 1.2 (1.1) | 6.4 (3.4) | 14.4 (8.1) | 9.6 (8.5) |
| Alcohol from beer in g/day at 3rd survey, mean (SD) | | 0 | 0.7 (0.6) | 2.3 (2.2) | 6.3 (6.1) | 3.9 (3.8) |
| Alcohol from spirits/liqueurs in g/day at 3rd survey, mean (SD) | | 0 | 0.4 (0.3) | 1.2 (1) | 2.8 (2.5) | 2.7 (2.6) |
| Years of education, n (%) | |  |  |  |  |  |
|  | ≤ 9 | 2 149 (29.3) | 7 960 (20.5) | 1 372 (13.1) | 210 (13) | 57 (16.7) |
|  | 10-12 | 2 404 (32.8) | 14 184 (36.5) | 3 396 (32.5) | 514 (31.9) | 104 (30.5) |
|  | ≥ 13 | 2 542 (34.7) | 15 643 (40.3) | 5 440 (52.1) | 836 (51.9) | 171 (50.2) |
|  | Missing | 237 (3.2) | 1 048 (2.7) | 238 (2.3) | 50 (3.1) | 9 (2.6) |
| Smoking status, n (%) | |  |  |  |  |  |
|  | Never | 4 094 (55.8) | 12 959 (33.4) | 2 452 (23.5) | 249 (15.5) | 47 (13.8) |
|  | Former | 1 464 (20) | 11 701 (30.1) | 3 564 (34.1) | 524 (32.6) | 106 (31.1) |
|  | Current | 1 635 (22.3) | 13 589 (35) | 4 299 (41.2) | 818 (50.8) | 187 (54.8) |
| . | Missing | 139 (1.9) | 586 (1.5) | 131 (1.3) | 19 (1.2) | 1 (0.3) |
| Self-rated health, n (%) | |  |  |  |  |  |
|  | Poor | 671 (9.2) | 1 812 (4.7) | 374 (3.6) | 74 (4.5) | 22 (6.5) |
|  | Good | 3 658 (49.9) | 19 623 (50.5) | 4 958 (47.5) | 804 (49.9) | 171 (50.2) |
|  | Excellent | 1 952 (26.6) | 11 854 (30.5) | 3 798 (36.4) | 583 (36.2) | 103 (30.2) |
|  | Missing | 1 051 (14.3) | 5 546 (14.3) | 1 316 (12.6) | 149 (9.3) | 45 (13.2) |
| BMI, mean (SD), kg/m2 | | 23.9 (4) | 23.2 (3.4) | 22.5 (2.8) | 22.3 (2.8) | 23.2 (3.6) |
| **Supplementary Table 4 Continued** | |  |  |  |  |  |
| BMI categories, n (%) | |  |  |  |  |  |
|  | <25 kg/m2 | 4 891 (66.7) | 29 232 (75.3) | 8 681 (83.1) | 1 383 (85.9) | 259 (76.0) |
|  | ≥25 kg/m2 | 2 227 (30.4) | 8 982 (23.1) | 1 641 (15.7) | 214 (13.3) | 78 (22.9) |
|  | Missing | 214 (2.9) | 621 (1.6) | 124 (1.2) | 13 (0.8) | 4 (1.2) |
| Physical activity level, n (%) | |  |  |  |  |  |
|  | Low | 3 271 (44.6) | 17 248 (44.4) | 4 708 (45.1) | 779 (48.4) | 172 (50.4) |
|  | Moderate-High | 3 143 (42.9) | 18 482 (47.6) | 5 189 (49.7) | 753 (46.8) | 131 (38.4) |
|  | Missing | 918 (12.5) | 3 105 (8.0) | 549 (5.3) | 78 (4.8) | 38 (11.1) |
| History of comorbidities, n (%) | |  |  |  |  |  |
|  | No | 6 637 (90.5) | 35 941 (92.6) | 9 788 (93.7) | 1 488 (92.4) | 302 (88.6) |
|  | Yes | 695 (9.5) | 2 894 (7.5) | 658 (6.3) | 122 (7.6) | 39 (11.4) |

Abbreviation: SD, standard deviation; BMI, body mass index.

Supplementary Table 5 Characteristics of women aged 50-70 years at enrolment according to trajectories of total alcohol consumption

|  | | **Non-drinker stable** | **Low stable** | **Light unstable** | **Light to high** | **Moderate decreasing** |
| --- | --- | --- | --- | --- | --- | --- |
| No (%) of women | | 4 212 (23.6) | 10 713 (60.1) | 2 160 (12.1) | 488 (2.7) | 245 (1.4) |
| Age, mean (SD), years | | 59.1 (5.9) | 57.5 (5.6) | 55.8 (5.2) | 56.7 (5.5) | 56.5 (5.4) |
| Total alcohol in g/day at 1st survey, mean (SD) | | 0 | 1.8 (1.6) | 7.7 (3.3) | 8.4 (3.9) | 17.7 (4.6) |
| Alcohol from wine in g/day at 1st survey, mean (SD) | | 0 | 0.9 (0.7) | 4.1 (2.8) | 5.1 (3.6) | 6.7 (3.8) |
| Alcohol from beer in g/day at 1st survey, mean (SD) | | 0 | 0.5 (0.4) | 1.9 (1.5) | 1.6 (1.3) | 5.6 (5.2) |
| Alcohol from spirits/liqueurs in g/day at 1st survey, mean (SD) | | 0 | 0.4 (0.4) | 1.7 (1.3) | 1.7 (1.5) | 5.4 (4.3) |
| Total Alcohol in g/day at 2nd survey, mean (SD) | | 0 | 1.7 (1.4) | 6.2 (3) | 12.5 (3.8) | 14.8 (6.1) |
| Alcohol from wine in g/day at 2nd survey, mean (SD) | | 0 | 0.9 (0.2) | 4.1 (3.1) | 8.2 (3.7) | 7 (4) |
| Alcohol from beer in g/day at 2nd survey, mean (SD) | | 0 | 0.4 (0.4) | 1.2 (1.2) | 2.3 (2.2) | 4 (3.9) |
| Alcohol from spirits/liqueurs in g/day at 2nd survey, mean (SD) | | 0 | 0.3 (0.2) | 0.9 (0.5) | 2 (2) | 3.8 (3.3) |
| Total alcohol in g/day at 3rd survey, mean (SD) | | 0 | 2.7 (2.3) | 9.3 (3.1) | 25.9 (4.9) | 13.6 (4.6) |
| Alcohol from wine in g/day at 3rd survey, mean (SD) | | 0 | 1.9 (1.9) | 6.8 (3.2) | 20(6.3) | 8.8 (4.6) |
| Alcohol from beer in g/day at 3rd survey, mean (SD) | | 0 | 0.5 (0.6) | 1.5 (1.4) | 3.1 (2.9) | 2.2 (2.1) |
| Alcohol from spirits/liqueurs in g/day at 3rd survey, mean (SD) | | 0 | 0.3 (0.3) | 1 (0.9) | 2.9 (2.3) | 2.6 (2.6) |
| Years of education, n (%) | |  |  |  |  |  |
|  | ≤ 9 | 2 206 (52.4) | 4 224 (39.4) | 460 (21.3) | 71 (14.6) | 40 (16.3) |
|  | 10-12 | 913 (21.7) | 3 019 (28.2) | 682 (31.6) | 122 (25.0) | 67 (27.4) |
|  | ≥ 13 | 592 (14.1) | 2 341 (21.9) | 822 (38.1) | 241 (49.4) | 118 (48.2) |
|  | Missing | 501 (11.9) | 1 129 (10.5) | 196 (9.1) | 54 (11.1) | 20 (8.2) |
| Smoking status, n (%) | |  |  |  |  |  |
|  | Never | 2 490 (59.1) | 4 198 (39.2) | 654 (30.3) | 120 (24.6) | 45 (18.4) |
|  | Former | 890 (21.1) | 3 475 (32.4) | 872 (40.4) | 212 (43.4) | 118 (48.2) |
|  | Current | 758 (18) | 2 935 (27.4) | 622 (28.8) | 155 (31.8) | 81 (33.1) |
|  | Missing | 74 (1.8) | 105 (1) | 12 (0.6) | 1 (0.2) | 1 (0.4) |
| Self-rated health, n (%) | |  |  |  |  |  |
|  | Poor | 454 (10.8) | 707 (6.6) | 92 (4.3) | 20 (4.1) | 4 (1.6) |
|  | Good | 2 418 (57.4) | 6 206 (57.9) | 1 088 (50.4) | 231 (47.3) | 120 (49) |
|  | Excellent | 636 (15.1) | 2 176 (20.3) | 647 (30) | 160 (32.8) | 81 (33.1) |
|  | Missing | 704 (16.7) | 1 624 (16.2) | 333 (15.4) | 77 (15.8) | 40 (16.3) |
| Supplementary Table 5 Continued | |  |  |  |  |  |
| BMI. mean (SD). kg/m2 | | 25.9 (4.2) | 25 (3.7) | 24.2 (3.2) | 23.9 (3.2) | 24.3 (3.7) |
| BMI categories, n (%) | |  |  |  |  |  |
|  | <25 kg/m2 | 1 898 (45.1) | 5 884 (54.9) | 1 422 (65.8) | 341 (69.9) | 161 (65.7) |
|  | ≥25 kg/m2 | 2 165 (51.4) | 4 622 (43.1) | 721 (33.4) | 142 (29.1) | 81 (33.1) |
|  | Missing | 149 (3.5) | 207 (1.9) | 17 (0.8) | 5 (1) | 3 (1.2) |
| Physical activity level, n (%) | |  |  |  |  |  |
|  | Low | 1 885 (44.8) | 5 267 (49.2) | 1 101 (51) | 256 (52.5) | 130 (53.1) |
|  | Moderate-High | 1 489 (35.4) | 4 178 (39) | 918 (42.5) | 200 (41) | 96 (39.2) |
|  | Missing | 838 (19.9) | 1 268 (11.8) | 141 (6.5) | 32 (6.6) | 19 (7.8) |
| History of comorbidities, n (%) | |  |  |  |  |  |
|  | No | 2 995 (71.1) | 8 400 (78.4) | 1 784 (82.6) | 409 (83.8) | 200 (81.6) |
|  | Yes | 1 217 (28.9) | 2 313 (21.6) | 376 (17.4) | 79 (16.2) | 45 (18.4) |

Supplementary Figure 16 Mean predicted trajectories with 95% confidence intervals (dash lines) of total alcohol consumption (g/day) when excluding women who died before returning the third questionnaire. The Norwegian Women and Cancer Study 1991-2011

1. Women aged 31-49 years at enrolment


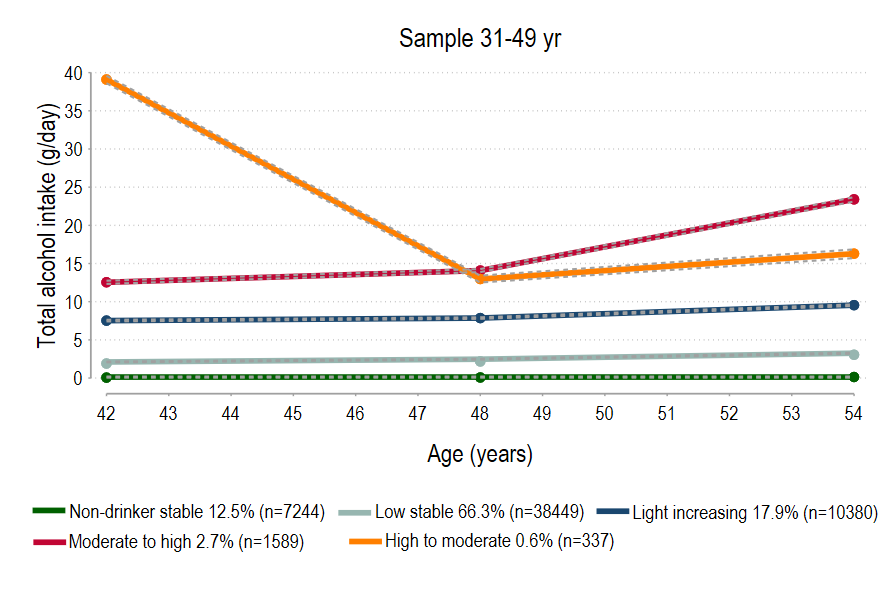


1. Women aged 50-70 years at enrolment


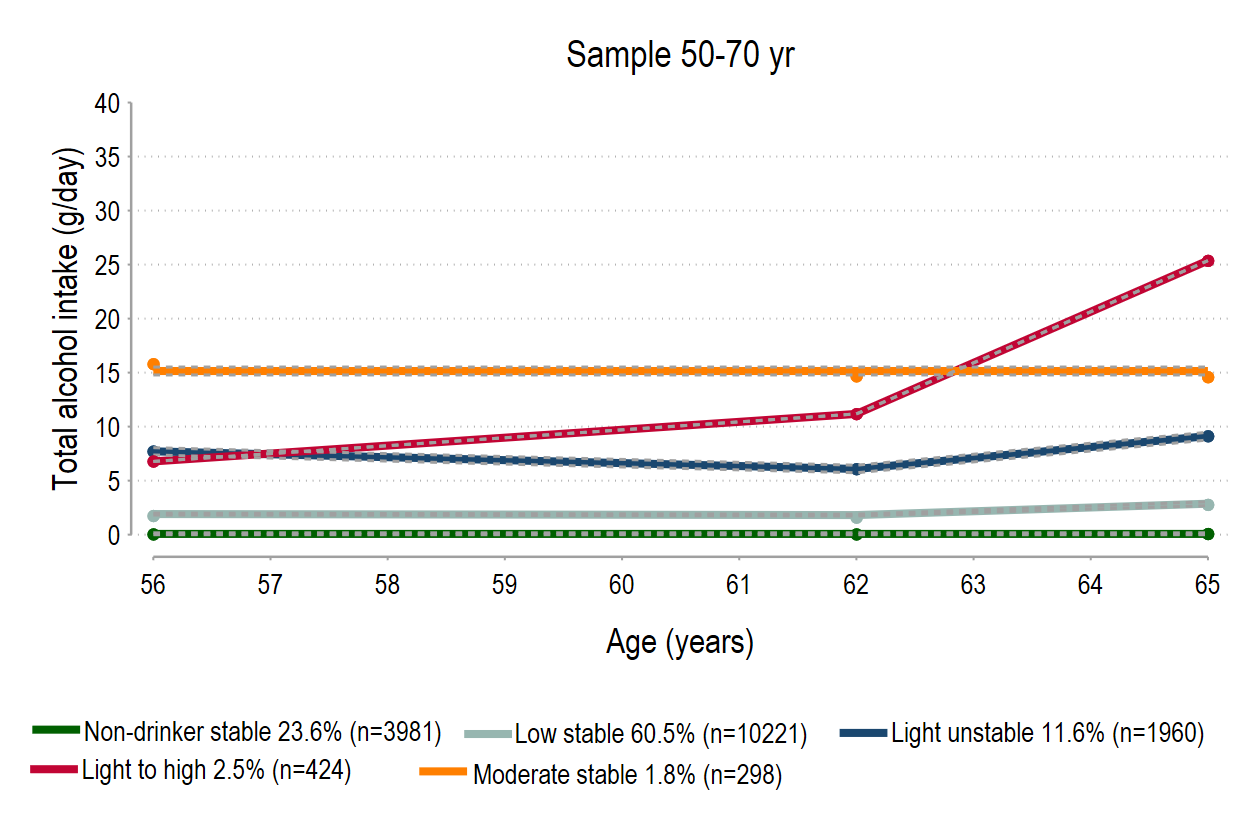


Supplementary Figure 17 Mean predicted trajectories with 95% confidence intervals (dash lines) of total alcohol consumption (g/day) in women aged 31-49 years at enrolment when excluding women for whom the recall period of alcohol data assessment matched with periods of pregnancy. The Norwegian Women and Cancer Study 1991-2011


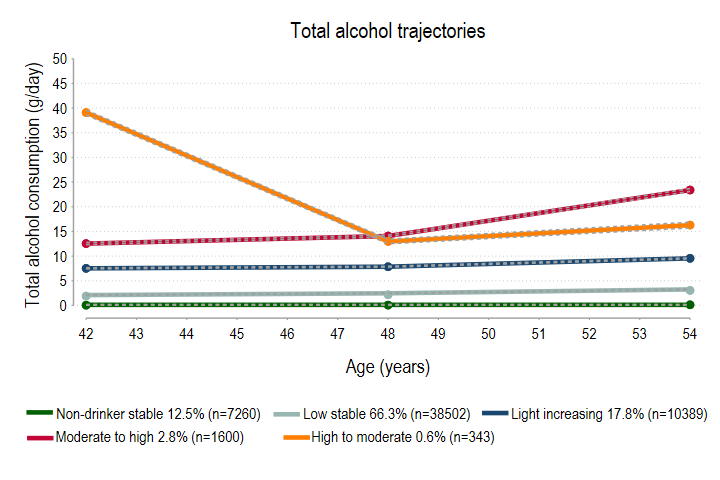


Supplementary Figure 18 Mean trajectories with 95% confidence intervals (dash lines) of total alcohol consumption (g/day) and of alcohol consumption from spirits when removing the consumption data for liqueurs. The Norwegian Women and Cancer Study 1991-2011

1. Women aged 31-49 years at enrolment


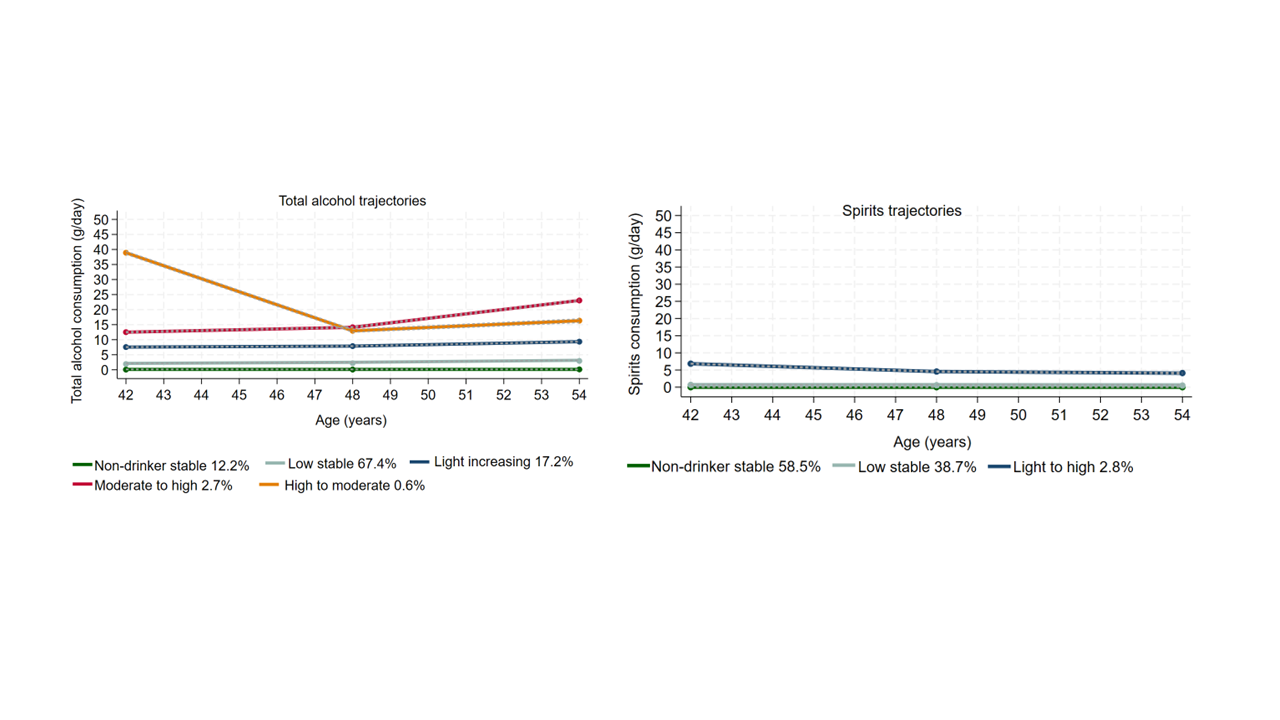


1. Women aged 50-70 years at enrolment


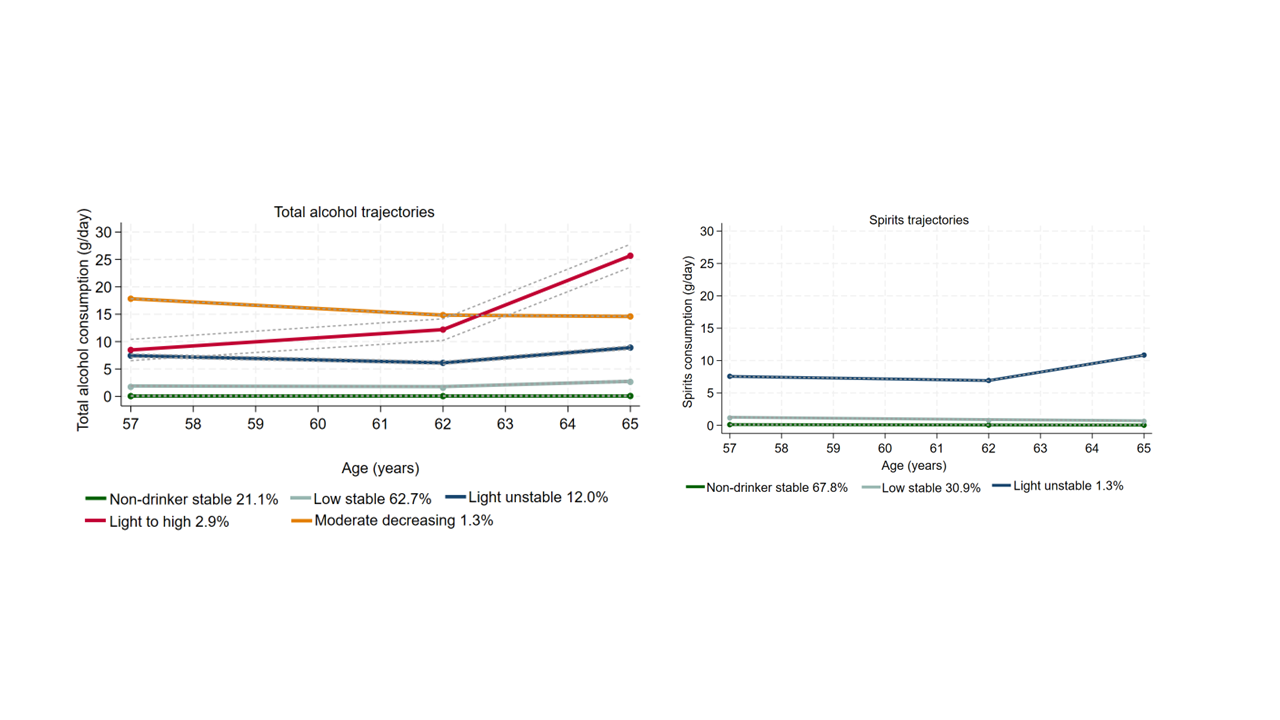


Supplementary references

1. Andruff H, Carraro N, Thompson A, Gaudreau P. Latent class growth modelling: A tutorial. pdfs.semanticscholar.org 2009;5(1):11–24. Available from: https://pdfs.semanticscholar.org/4591/833f7f058408f0587f83ea5e9efbe7586f78.pdf. Accessed 12 Jul 2023.

2. Nagin DS, Odgers CL. Group-based trajectory modeling in clinical research. Annu Rev Clin Psychol 2010;6:109–138.

3. Nagin DS, Jones BL, Passos VL, Tremblay RE. Group-based multi-trajectory modeling. Stat Methods Med Res 2018;27(7):2015–2023.

4. Bassett JK, MacInnis RJ, Yang Y, et al. Alcohol intake trajectories during the life course and risk of alcohol-related cancer: A prospective cohort study. Int J cancer 2022;151(1):56–66.

Stata codes of the final GBTM models

//Final GBTM model and their plots in the younger subcohort//

**For total alcohol consumption**

traj, var(alco_intake_grams_*) indep(age_*) model(cnorm) min(0) max(100) order(0 2 2 2 2)

trajplot, xtitle(Age (years)) ytitle(Total alcohol consumption (g/day)) xlabel(42(1)54) ylabel(0(5)50 ) ci

**For wine consumption**

traj, var(wine_intake_grams_*) indep(t_*) model(cnorm) min(0) max(60) order(1 2 1 2 2)

trajplot, xtitle(Age (years)) ytitle(Wine consumption (g/day)) xlabel(42(1)54) ylabel(0(5)50) ci

**For beer consumption**

traj, var(beer_intake_grams_*) indep(t_*) model(cnorm) min(0) max(80) order(1 0 0 2)

trajplot, xtitle(Age (years)) ytitle(Beer consumption (g/day)) xlabel(42(1)54) ylabel(0(5)50) ci

** For spirits and liqueurs consumption**

traj, var(spir_liq_intake_grams_*) indep(t_*) model(cnorm) min(0) max(60) order(0 2 2)

trajplot, xtitle(Age (years)) ytitle(Spirits/liqueurs consumption (g/day)) xlabel(42(1)54) ylabel (0(5)50) ci

//Final GBTM model and their plots in the younger subcohort//

**For total alcohol consumption**

traj, var(alco_intake_grams_*) indep(t_*) model(cnorm) min(0) max(50) order(0 2 2 2 1)

trajplot, xtitle(Age (years)) ytitle(Total alcohol consumption (g/day)) xlabel(57(1)65) ylabel(0(5)30 ) ci

**For wine consumption**

traj, var(wine_intake_grams_*) indep(t_*) model(cnorm) min(0) max(25) order(1 2 2 2 1)

trajplot, xtitle(Age (years)) ytitle(Wine consumption (g/day)) xlabel(57(1)65) ylabel(0(5)30) ci

**For beer consumption**

traj, var(beer_intake_grams_*) indep(t_*) model(cnorm) min(0) max(40) order(1 1 2)

trajplot, xtitle(Age (years)) ytitle(Beer consumption (g/day)) xlabel(57(1)65) ylabel(0(5)30) ci

** For spirits and liqueurs consumption**

traj, var(spir_liq_intake_grams_*) indep(t_*) model(cnorm) min(0) max(30) order(1 1 2)

trajplot, xtitle(Age (years)) ytitle(Spirits/liqueurs consumption (g/day)) xlabel(57(1)65) ylabel (0(5)30)

***Fit statistics of the final GBTM models were printed out using the following Stata codes created by Anrew P. Wheeler. These codes can be found on the creator's website: https://andrewpwheeler.com/2016/10/06/group-based-trajectory-models-in-stata-some-graphs-and-fit-statistics***

program summary_table_procTraj

preserve

*updating code to drop missing assigned observations

drop if missing(_traj_Group)

*now lets look at the average posterior probability

gen Mp = 0

foreach i of varlist _traj_ProbG* {

replace Mp = `i' if `i' > Mp

}

sort _traj_Group

*and the odds of correct classification

by _traj_Group: gen countG = _N

by _traj_Group: egen groupAPP = mean(Mp)

by _traj_Group: gen counter = _n

gen n = groupAPP/(1 - groupAPP)

gen p = countG/ _N

gen d = p/(1-p)

gen occ = n/d

*Estimated proportion for each group

scalar c = 0

gen TotProb = 0

foreach i of varlist _traj_ProbG* {

scalar c = c + 1

quietly summarize `i'

replace TotProb = r(sum)/ _N if _traj_Group == c

}

gen d_pp = TotProb/(1 - TotProb)

gen occ_pp = n/d_pp

*This displays the group number [_traj_~p],

*the count per group (based on the max post prob), [countG]

*the average posterior probability for each group, [groupAPP]

*the odds of correct classification (based on the max post prob group assignment), [occ]

*the odds of correct classification (based on the weighted post. prob), [occ_pp]

*and the observed probability of groups versus the probability [p]

*based on the posterior probabilities [TotProb]

list _traj_Group countG groupAPP occ occ_pp p TotProb if counter == 1

restore

end
